# In-feed provision of binding proteins sustains piglet gut health and mitigates ETEC-induced post-weaning diarrhea

**DOI:** 10.1186/s40104-025-01209-6

**Published:** 2025-06-02

**Authors:** Jiajia Xu, Melania Andrani, Rikke Brødsgaard Kjærup, Tina Sørensen Dalgaard, Carsten Eriksen, Andreas Hougaard Laustsen, Susanne Brix, Sandra Wingaard Thrane, Nuria Canibe

**Affiliations:** 1 Department of Animal and Veterinary Sciences, Aarhus University, Tjele, Denmark; 2 Department of Veterinary Science, University of Parma, Parma, Italy; 3 Department of Biotechnology and Biomedicine, Technical University of Denmark, Kongens Lyngby, Denmark; 4 Bactolife A/S, Rønnegade 8, Copenhagen Ø, Denmark

**Keywords:** Antimicrobial alternatives, Binding proteins, Enterotoxigenic *E. coli*, Feed additive, Gut health, Piglets, Post-weaning diarrhea, Single-domain antibodies

## Abstract

**Background:**

Post-weaning diarrhea (PWD) in piglets, often caused by F4^+^ enterotoxigenic *Escherichia coli* (ETEC), poses significant challenges in pig production. Traditional solutions like antibiotics and zinc oxide face increasing restrictions due to growing concerns over antibiotic resistance and environmental sustainability. This study investigates the application of bivalent heavy chain variable domain (V_H_H) constructs (BL1.2 and BL2.2) targeting ETEC virulence factors, administered in feed to mitigate ETEC-induced PWD in weaned piglets.

**Results:**

The supplementation of BL1.2 and BL2.2 in both mash and pelleted feed significantly reduced the diarrhea incidence and fecal shedding of F4^+^ ETEC in challenged piglets. Pelleted feed containing V_H_H constructs helped to preserve gut barrier integrity by maintaining levels of the tight junction protein occludin in the small intestine. Additionally, the constructs maintained blood granulocyte counts at a similar level to the non-challenged control group, including neutrophils, and ameliorated the acute phase protein response after challenge. Notably, even at low feed intake immediately after weaning, V_H_H constructs helped maintain piglet health by mitigating ETEC-induced inflammation and the resulting diarrhea.

**Conclusions:**

Our findings demonstrated that using V_H_H constructs as feed additives could serve as an effective strategy to help manage ETEC-associated PWD, by reducing F4^+^ ETEC gut colonization and supporting gut barrier function of weaned piglets. The high stability of these V_H_H constructs supports their incorporation into industrial feed manufacturing processes, offering a more sustainable preventive strategy compared to traditional antimicrobial interventions, which could contribute to sustainable farming practices.

**Supplementary Information:**

The online version contains supplementary material available at 10.1186/s40104-025-01209-6.

## Background

The post-weaning period in piglets is critically challenged by enterotoxigenic *Escherichia coli* (ETEC) infections, which are a leading cause of post-weaning diarrhea (PWD) [1]. This condition undermines the health and growth of piglets, imposing significant economic impairments on pig farming. With the growing concerns over antibiotic resistance and environmental sustainability, the reliance on traditional treatments like antibiotics and zinc oxide is increasingly problematic [2–6]. Antibiotic treatment for controlling gastrointestinal infections can also disrupt the gut commensal microbiota, leading to dysbiosis, and as a consequence, to other detrimental effects like damage of the epithelial gut barrier [7]. This context highlights the pressing need for innovative and preventive solutions to effectively manage ETEC-related health issues in weaned piglets, changing the focus from treatment of infections to enhancing resilience and promoting gut health.

ETEC strains, particularly those expressing F4 and F18 fimbriae, are present across industrial farms globally and are closely associated with the onset of PWD [8, 9]. The pathogenicity of ETEC is primarily attributed to its ability to adhere to the intestinal epithelium through specific surface proteins, including the aforementioned fimbriae [10, 11]. The initial step of attachment in this adhesion process is crucial for colonization and subsequent infection, making the fimbriae relevant targets for intervention strategies aiming to reduce the proliferation of ETEC in piglets [12]. In addition to their fimbriae, ETEC produce enterotoxins such as the heat‐labile enterotoxin (LT) and the heat‐stable enterotoxins (STa and STb) [13]. These toxins exert their action by disturbing fluid homeostasis in the intestine, which leads to the onset of diarrhea [8].

Previously, we have shown that oral supplementation of a bivalent V_H_H construct specific for the F4 fimbriae, i.e., BL1.2, can reduce the proliferation of F4^+^ ETEC in vivo [14]. We have also shown that oral administration (by gavage) of BL1.2 together with another LT-specific bivalent V_H_H construct, BL2.2, can reduce F4^+^ ETEC proliferation and support the maturation of a healthy gut microbiota after weaning in F4^+^ ETEC-challenged piglets [15]. Combined, these former studies thus indicate that oral administration of bivalent V_H_H constructs targeting the key pathogenic factors of F4^+^ ETEC could be a beneficial strategy to mitigate PWD in industrial pig production. However, for such a mitigation strategy to be industrially applicable, it is important that the mode of administration is compatible with farming practices, and that the bivalent V_H_H constructs can withstand industrial feed manufacturing processes. Therefore, we set out to investigate whether BL1.2 and BL2.2 retain their in vivo functionality when provided ad libitum in feed, firstly when admixed into mash feed, and secondly when included in a standard pelleted feed. Moreover, we aimed to investigate whether the expected reduction in F4^+^ ETEC proliferation in the piglet gut would translate into reduced inflammation and improved gut health status in F4^+^ ETEC-challenged piglets.

## Materials and methods

### Animals and housing

This study was performed at Aarhus University, Department of Animal and Veterinary Sciences (AU Viborg, Denmark). Animal care and housing were in accordance with Danish laws and regulations governing the humane care and use of animals in research.

In each experiment, piglets from five sows (Duroc × Landrace × Yorkshire mated with Norsvin Landrace boar, parity 4 in Exp. A, parity 2 in Exp. B) confirmed to be homozygous carriers of the dominant gene (*MUC4* gene) coding for intestinal ETEC F4 fimbriae receptors using competitive allele specific PCR (KASP) (VHL Genetics, Netherlands) were used. Sows were fed a standard Danish sow diet and with amounts according to Danish norms [16]. At weaning (24–25 days old in Exp. A and 26–27 days old in Exp. B), 30 piglets of both sexes were randomly allocated into three experimental groups, each housed in a separate room, with two littermates in each pen, resulting in 10 piglets per group. The two sibling piglets were housed in pens (215 cm × 110 cm), with 75 cm × 110 cm slatted floor and 140 cm × 110 cm concrete floor, with floor heating and partial coverage. To prevent physical contact between piglets from different pens, an empty pen was allowed between pens housing pigs. Feed and water were provided ad libitum. No bedding was allowed in the pen, but each pen was equipped with a rope to help satisfy the rooting behavior of the piglets. In Exp. A, the room temperature was maintained at 25.0 °C post-weaning, then lowered to 23.7 °C during the third week. In Exp. B, the room temperature remained at 25.0 °C. Humidity levels were consistently kept at 55.7% in Exp. A and 59.2% in Exp. B.

### Experimental groups and procedures

**Fig. 1:**
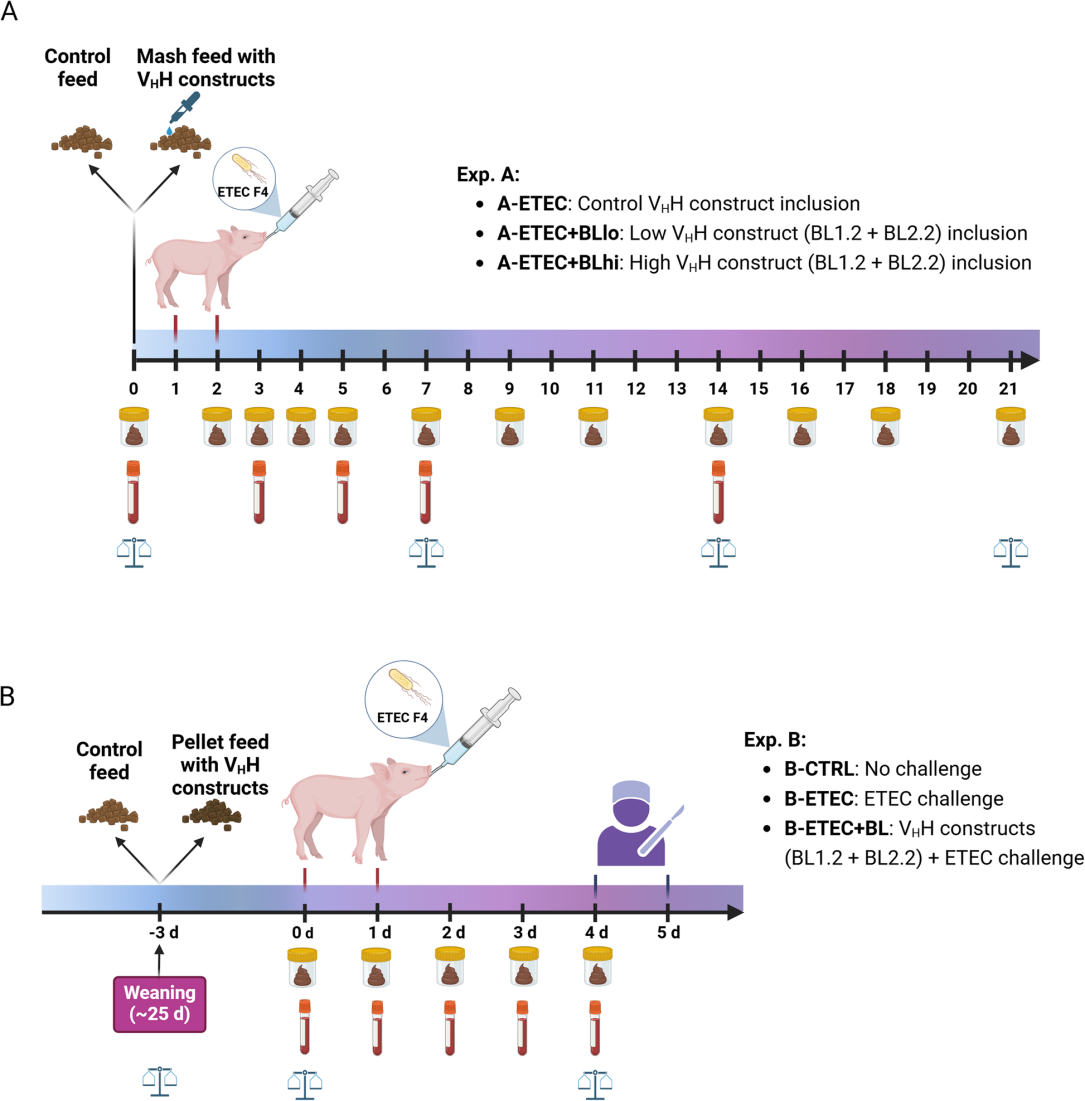
Experimental design and timeline for Exp. A and B.** A** Experiment A included three groups of piglets (*n* = 30) that were all subjected to an ETEC challenge on d 1 and 2. A-ETEC piglets were fed feed containing non-binding V_H_H constructs, whereas A-ETEC + BLlo were fed feed containing 50 mg/kg of the bivalent V_H_H constructs, and A-ETEC + BLhi were fed feed containing 150 mg/kg of the bivalent V_H_H constructs. The experiment was run over 21 d, during which the piglets were weighed on d 0, 7, 14, and 21, blood samples were taken on d 0, 3, 5, 7, and 14, and stool samples were collected on d 0, 2, 3, 4, 5, 7, 9, 11, 14, 16, 18, and 21. **B** Experiment B included three groups of piglets (*n* = 30), where B-CTRL piglets were not challenged, fed a control diet; B-ETEC were challenged with ETEC, fed a control diet; and B-ETEC + BL were challenged with ETEC, fed feed containing 52.5 mg/kg of the bivalent V_H_H constructs. The experiment was run over 9 d, during which the piglets were weighed on d −3, 0, and 4, and blood and stool samples were collected on d 0, 1, 2, 3, and 4. Created in BioRender [17]

The experimental design and timeline are shown in Fig. 1. The ETEC strain *E. coli* O149:F4 (9910045-1, Aarhus University), which has the genes for and produces STb, LT, and adhesin F4ac, was used to challenge the piglets. This strain, which was isolated by the Danish Veterinary Institute (Copenhagen, Denmark), is routinely used in our research group and was used in two previous in vivo studies where the efficacy of BL1.2 and BL2.2 was tested [14, 15].

To ensure the safety of the experimental setup, strict biosecurity measures were followed. Personnel were required to wear shoe covers and single-use protective suits when entering the experimental room. Gloves, plastic sleeves, aprons, and boots were replaced when entering different rooms. The room with the non-challenged group was handled first.

#### Exp. A

A standard weaner diet was formulated (Table S1) and different V_H_H constructs were added at different inclusion rates. The diet was fed as mash. Three experimental groups were included: A-ETEC (*n* = 10): 50 mg non-binding V_H_H constructs/kg feed; A-ETEC + BLlo (*n* = 10): low V_H_H constructs supplementation, i.e., 50 mg of each of the V_H_H constructs (BL1.2 and BL2.2)/kg feed; A-ETEC + BLhi (*n* = 10): high V_H_H constructs supplementation, i.e., 150 mg of each of the V_H_H constructs (BL1.2 and BL2.2)/kg feed. The inclusion rate of V_H_H constructs in the feed was determined based on expected feed intake data from a previous trial with a similar setup, where piglets consumed ca. 100 g/pig on the first day post-weaning. This rate was designed to ensure that piglets received a sufficient amount of the V_H_H constructs during the critical days immediately after weaning, with the goal of reaching 5 mg/V_H_H/pig/d for A-ETEC + BLlo group and 15 mg/V_H_H/pig/d for the A-ETEC + BLhi group. The V_H_H constructs (BL1.2 and BL2.2) were freeze-dried to maintain integrity and standardization, and then mixed in the feed and fed during the entire experiment period. On d 1 and 2 after weaning, all piglets were orally inoculated with 5 mL of 10^9^ CFU ETEC/mL to induce mild diarrhea. Exp. A lasted 21 d (Fig. 1A).

#### Exp. B

A standard weaner diet was formulated (Table S2), and V_H_H constructs were added before pelleting. The V_H_H constructs BL1.2 and BL2.2 were coated in granulates with a particle size range of 300–1200 µm (median diameter of particles = 600 µm) and subsequently pelleted into pellets measuring 10 mm × 3.5 mm at 81 °C, resulting in an inclusion rate of 52.5 mg of each of the V_H_H constructs (BL1.2 and BL2.2)/kg pelleted feed. The inclusion of V_H_H constructs was calculated using feed intake from Exp. A, aiming at 15 mg/V_H_H/pig/d. Three experimental groups were included: B-CTRL (*n* = 10): fed with a control diet without being challenged; B-ETEC (*n* = 10): fed with the control diet and challenged with F4^+^ ETEC; B-ETEC + BL (*n* = 10): fed with V_H_H constructs (52.5 mg BL1.2 and 52.5 mg BL2.2/kg) pelleted in the feed and challenged with F4^+^ ETEC. Since weaning (d −3), all piglets were fed according to the different experimental groups. On d 0 and 1, piglets in the B-ETEC + BL and B-ETEC groups were challenged with F4^+^ ETEC (9 mL of 10^9^ CFU/mL on d 0 and 5.5 mL of 10^9^ CFU/mL on d 1), piglets in the B-CTRL group were orally given the same volume of saline solution. On d 4 and 5, all piglets were sacrificed. Exp. B lasted 9 d (Fig. 1B).

### Production and stability test of VHH constructs

#### Production of VHH constructs

As in our previous studies [14, 15], for the in vivo challenge study, untagged BL1.2 (27.92 kDa) and BL2.2 (28.04 kDa) constructs were produced at Novonesis laboratories (Bagsværd, Denmark).

#### Freeze drying of VHH constructs (Exp. A)

Protein ferments (BL1.2, BL2.2, or control) were frozen at −80 °C and freeze-dried for 24 h. All samples dried well into a uniform powder.

#### Feed granulate formulation and stability (Exp. B)

The V_H_H fermentation supernatant was formulated into a standard feed granulate with a particle size of 300–1200 µm (D50 = 600 µm), compatible with feed production. The stability of the granulate was measured by taking 3 g of each (BL1.2 and BL2.2) and exposing them to different temperatures to mimic a steam and extruder pelleting process as follows. The tested temperatures were 90, 150, and 300 s at −18°C, 300 s at 75 °C, 150 s at 75 °C, and 90 s at 95 °C. For all temperatures above 0 °C, relative humidity was 95% when testing. Retained protein integrity was examined from two separate samples of each granulate using SDS-PAGE and ELISA (see below).

#### Feed pelleting

Pelleted feed was produced by the Danish Technological Institute, using a mini feed milling plant designed for small feed batch production under industrially relevant conditions. The plant can handle 80–300 kg of meal, and the nominal capacity is 300 kg/h. A total of 200 kg feed was produced (1/3 including V_H_H constructs and 2/3 control).

V_H_H-containing granulates were added to the meal together with the premix (a mixture of vitamins, minerals, trace elements, and other feed additives that are incorporated at levels between 0.2% and 0.5%). The feed pellets were produced according to standard piglet feed process, as 3 mm (diameter) × 35 mm pellets using hammer milling, with maximal meal temperature set to 80.7–81.2 °C and 4.2% steam with a retention time of 30 s.

#### SDS-PAGE of granulates

All samples for SDS-PAGE were mixed with 4 × LDS buffer (Genscript Biotech, Piscataway, NJ, USA) containing 50 mmol/L dithiothreitol (DTT0029). Samples were boiled and loaded on NuPage 4–12% Bis–Tris protein gels (Invitrogen, USA) together with a PageRuler prestained protein ladder (Thermo Fisher Scientific, Foster City, USA). The gels were run in NuPage MES SDS running buffer (Invitrogen, USA) at 90 V for 15 min followed by 150 V for 45 min. Gels were stained using Coomassie Brilliant Blue R-250 (Thermo Fisher Scientific, Foster City, USA).

#### Binding activity of VHH constructs by ELISA determinations

All ELISA assays were performed on Maxisorp plates (Nunc, Thermo Fisher Scientific). Binding analyses were performed at least in triplicates. Maxisorp plates were coated with 4 and 2 μg/mL FaeG or LT-B antigen, respectively. The plates were washed and blocked with 10% skim milk powder, 0.02% Tween in phosphate buffered saline (PBS) (10% M-PBS). BL1.2 and BL2.2 granulates were resuspended in PBS (1% w/v) and diluted 1:2,000 in 1% skim milk powder, 0.02% Tween in PBS (1% M-PBS), and added to coated Maxisorp plates. Bound protein was detected using Protein A conjugated to horseradish peroxidase (HRP) (GenScript, USA) diluted 1:5,000 (BL1.2) or 1:20,000 (BL2.2) in 1% M-PBS. Plates were developed by incubating with 3,3′,5,5′-tetramethylbenzidine-peroxide solution, and the reaction was stopped with 2 mol/L H_3_PO_4_. Absorbance was measured at 450 nm.

### Registrations and sampling

#### Exp. A

Individual body weight of all pigs was registered at weaning and weekly during the experiment. Feed intake per pen was registered daily. Individual fecal samples from all pigs were collected directly from the rectum using a gel-lubricated glove on d 0 (weaning day), d 2–5, and on d 7, 9, 11, 14, 16, 18, and 21 post-weaning. Feces samples were evaluated based on a seven-category scale (1: Hard, dry, lumpy. 2: Firm. 3: Soft but formable. 4: Soft and liquid. 5: Watery, dark. 6: Watery, yellow. 7: Yellow, foaming) as described by Carstensen et al. [18], with score 4–7 classified as diarrhea. Fecal samples were divided into three subsamples: one for bacteria enumeration (immediately performed), one for dry matter (DM) determination (stored at −20 °C), and one for DNA extraction followed by quantitative real-time polymerase chain reaction (qPCR) (stored at −80 °C). When the sample amount was insufficient for all three subsamples, the subsample used for DNA extraction was prioritized. Blood samples were obtained from all pigs from the jugular vein on the weaning day, i.e., d 0, and 3, 5, 7, and 14 post-weaning. Plasma samples were obtained by centrifugation (3,000 × *g*, 4 °C, 10 min) and stored at −20 °C until C-reactive protein (CRP) and haptoglobin analysis were performed.

#### Exp. B

Individual body weight of all pigs was registered on the day of weaning (d −3 relative to the first challenge day), d 0 (first challenge day), and d 4 after the first challenge. Daily feed consumption per pen was registered during the whole period. Rectal temperature was measured daily from the first challenge day. Fecal samples were obtained from all pigs from d 0 to d 4 post-challenge, following the same procedure as in Exp. A. Individual fecal samples were collected, scored according to the same scale as in Exp. A, and divided into two subsamples: one for DM determination (stored at −20 °C) and one for DNA extraction for ETEC F4 fimbriae (*faeG* gene), LT toxin (*eltB* gene), and STb toxin (*est-II* gene) by qPCR (stored at −80 °C). Blood samples were obtained from the jugular vein in BD Vacutainer® EDTA tubes (Becton Dickinson, USA) and immediately placed on ice for analysis of leukocyte and lymphocyte counts by flow cytometry, and complete blood cell counts using a hematology analyzer (IDEXX ProCyte Dx^®^, IDEXX Laboratories Inc., USA). Plasma was obtained by centrifugation (3,000 × *g*, 4 °C, 10 min), collected and stored in polyethylene tubes at −20 °C until the determination of CRP, haptoglobin, and pig major acute-phase protein (Pig-MAP) concentration was performed.

On d 4 and 5, five pigs per group were sacrificed each day, and digesta, mucosa, and tissue samples were obtained. The pigs were euthanized using a captive bolt gun followed by exsanguination, after which the gastrointestinal tract was removed and divided into stomach (Sto), two equally long segments of the small intestine (SI1, SI2), cecum (Cae), and two equally long segments of the colon (Co1, Co2). Digesta pH as well as digesta weight of each segment were measured and recorded. For determination of DM content, digesta samples from all segments were transferred into pre-weighed plastic containers, the containers with samples were weighed and frozen at −20 °C until freeze-drying was performed. Small intestinal tissue samples were taken at 50% (SI50) and 90% (SI90) of the small intestine length. After identifying the locations SI50 and SI90, samples were collected starting from the distal to the proximal gastrointestinal tract, while avoiding Peyer’s patches, in the following order. For intestinal morphology measurements, a 5-cm epithelium sample was taken and kept in 10% formalin. For gene expression analysis, i.e., toll-like receptor 2 (*TLR2*), *TLR4*, interleukin-10 (*IL10*), *IL17*, *IL22*, *IL23A*, transforming growth factor beta (*TGFb*), glyceraldehyde-3-phosphate dehydrogenase (*GAPDH*), and actin beta (*ACTB*), a 2-cm sample was taken and rinsed using PBS and transferred into RNAlater and stored at −20 °C. For tight junction protein analysis by western blot, a 2-cm sample was collected, cut into pieces, and transferred to cryotubes and snap-frozen in liquid nitrogen prior to storage at −80 °C. For ETEC F4 fimbriae (*faeG* gene) quantification, a mucosal sample derived from 30 cm of intestine was obtained by gently scraping off the mucosa from the epithelial layer using a sterile microscope slide on a frozen plate and snap-frozen in liquid nitrogen prior to storage at −80 °C.

### Analytical methods

#### Dry matter content and qPCR

The DM content of feces and digesta samples was determined by freeze-drying. The methodology followed to determine F4^+^ ETEC counts in feces, and the procedure for DNA extraction and qPCR to quantify ETEC F4 fimbriae (*faeG* gene) in feces, gut digesta, and mucosa were as described by Xu et al. [19], except for the amount of sample used for DNA extraction (see below). Briefly, fecal samples were homogenized, serially diluted, and plated on blood agar for hemolytic bacteria enumeration. F4^+^ ETEC serotyping was performed on five randomly selected colonies per plate using the slide agglutination test with type-specific antisera (SSI Diagnostica A/S, Hillerød, Denmark). DNA in feces, digesta, and mucosa samples was extracted using the NucleoSpin 96 DNA Stool kit (Macherey–Nagel, Germany), with modifications for the different sample types: 50 mg feces, mucosa, Cae, Co1, and Co2 digesta samples, and 200 mg Sto, SI1, and SI2 digesta samples were weighed. The qPCR conditions and primer sequences used were identical to those described by Xu et al. [19]. The cycle threshold (Ct) cut-off values were 30 for F4 fimbriae (*faeG* gene), 31 for LT toxin (*eltB* gene), and 30 for STb toxin (*est-II* gene), and these were determined as the limit of quantification (LOQ) for the assay.

#### Intestinal morphology

After 48 h in 10% formalin, the tissue samples from SI50 and SI90 were dehydrated and embedded in paraffin wax as described by Engelsmann et al. [20]. A slide was prepared from each sample containing 5–6 sections cut with a thickness of 4 µm. All slides were stained with hematoxylin and eosin (H&E). The height of villi and depth of crypts were assessed by measuring 15 villi and 15 corresponding crypts at × 20 objective magnification for each sample. The villi and crypt densities were measured at × 20 objective magnification and were defined as the number of crypts or villi present over a defined distance (560 µm) across the luminal side of the mucosa.

#### Gene expression

Total RNA was extracted from the small intestinal epithelium using the NucleoSpin RNA kit (740955, Macherey–Nagel, Germany) following the user manual with some adjustments. Approximately 15–20 mg of tissue was weighed into EP tubes, to which one 5 mm stainless steel bead (69989, Qiagen) and 600 µL lysis buffer (RA1 mixed with 1% β-mercaptoethanol) were added. The samples were then homogenized using a pre-cooled TissueLyser LT (Qiagen, Germany) at 50 oscillations/s for 2.5 min. Subsequently, 350 µL of the homogenized tissue was used for RNA extraction. RNA was eluted twice with 30 µL of RNase-free water followed by centrifugation (11,000 × *g*, 1 min). RNA concentration and purity were assessed using a NanoDrop® ND-1000 Spectrophotometer. The RNA samples were diluted into 40 ng/µL by adding RNase-free water. From the six samples with the highest RNA concentration, replicates were made for later use as standards in the RT-PCR step.

The cDNA samples were synthesized from diluted RNA samples using High-Capacity cDNA Archive kit (4,368814, Applied Biosystems). Briefly, for each sample, 15 µL 2 × RT master mix and 15 µL total RNA (40 ng/µL) were added into the strip PCR tubes placed on ice, and then vortexed and spun down shortly. These mixtures were then amplified using the following program in a PCR machine (Swift MaxPro, Holm & Halby, Denmark): 25 °C for 10 min, 37 °C for 120 min, followed by a hold at 4 °C. This yielded 30 µL of cDNA with an expected concentration of 20 ng/µL. For standards (six samples with the highest RNA concentration) preparation, 30 µL of RNase-free water was added to achieve 10 ng/µL cDNA. Then, the six samples were pooled. For other samples, 60 µL RNase-free water was added to achieve 6.67 ng/µL cDNA. These cDNA samples were stored at −20 °C for subsequent RT-qPCR analysis. For qPCR standards, the six pooled cDNA samples were serially diluted four-fold.

Predesigned TaqMan Gene Expression Assays (Thermo Fisher Scientific) were used for *TLR2* (Ss03381278_u1), *TLR4* (Ss04956023_s1), *IL10* (Ss03382372_u1), *IL17A* (Ss03391803_m1), *IL22* (Ss03373919_m1), *IL23A* (Ss03648973_m1), *TGFb* (Ss03382325_u1), *GAPDH* (Ss03375629_u1), and *ACTB* (Ss03376563_uH) gene expression analysis. Standard curves were made in triplicate with serial four-fold dilutions from the pool of cDNA. RT-qPCR was performed on the Applied Biosystems ViiA7 real-time PCR system (Thermo Fisher Scientific) using MicroAmp Optical 384-well reaction plates (Applied Biosystems) and all reactions were done in duplicate. A final volume reaction of 10 μL containing 5 μL Taqman Fast Advanced master mix (4444963, Applied Biosystems), 0.5 μL TaqMan Gene Expression Assays, 2 μL cDNA, and 2.5 μL water was used. *GAPDH* and *ACTB* were used as reference genes and were confirmed to be suitable as reference genes using GeNorm. The temperature profile was as follows: 20 s at 95 °C, 40 cycles of 1 s denaturation at 95 °C, and 20 s annealing at 60 °C.

#### Gut epithelial tight junctions

Tissue samples (approximately 60–90 mg) were homogenized in liquid nitrogen using a mortar and pestle. For each 5 mg of powdered sample, 240 μL lysis buffer (10 mmol/L Tris–HCl at pH 8, 10 mmol/L NaCl, 3 mmol/L MgCl_2_, 0.1% SDS, 0.1% Triton X-100, 0.5 mmol/L EDTA) containing a protease inhibitor mix (10 µg/mL APMSF, 0.5 µg/mL leupeptin, 0.7 µg/mL pepstatin A) was added to tubes placed on ice, vortexed twice for 10 s and kept on ice for 10 min. After centrifugation (4 °C, 30 min, 15,000 × *g*), the supernatant was collected, and the protein level was measured using a Pierce BCA Protein assay kit (Thermo Fisher Scientific, USA). The amount of protein load for each sample was 18 µg. To normalize the volume across samples, lysis buffer was added. After this, samples were boiled at 100 °C for 5 min and then stored at −20 °C until the electrophoresis process the following day. Prior to electrophoresis, 5 × Laemmli Buffer (containing bromophenol blue, SDS, glycerol, Tris-HCl) was added to the samples and mixed thoroughly. Proteins were then separated by 10% SDS-PAGE in Tris buffer, using Hoefer Mighty Small™ II Mini Vertical Electrophoresis Systems (Amersham Biosciences Inc, UK). A PageRuler pre-stained protein ladder with a range of 10–250 kDa (2661, Thermo Fisher Scientific, USA) was used as the molecular weight standard. Following electrophoresis, the protein samples were transferred onto a Hybond^®^-P PVDF membrane (Amersham Pharmacia Biotech, Italy) using Hoefer TE22 Mighty Small Transfer Tank (Amersham Biosciences Inc, UK). The membrane was first immersed in blocking buffer (5% skim milk, 1 × PBS with 0.05% Tween20 (PBST)) for 1 h at room temperature, then incubated using the same buffer containing a specific primary antibody targeting either: Occludin (OCLN) 1:1,000 (66378-1-lg, Proteintech, USA) or β-actin 1:1,500 (4970S, Cell Signaling, USA) at 4 °C overnight. After repeated washes with PBST, 1:50,000 secondary antibodies were applied for 1 h at room temperature. Specifically HRP-conjugated goat anti-mouse IgG (31430, Thermo Fisher Scientific, USA) was used for OCLN and HRP-conjugated goat anti-rabbit IgG (31460, Thermo Fisher Scientific, USA) for β-actin. Following this, the membrane was washed twice using PBST. For luminescence analysis, 8 μL Super Signal West Pico PLUS Chemiluminescent Substrate (34580; Thermo Fisher Scientific, USA) was applied. The band signal intensities were detected using ChemiDoc™ MP Imaging System (Bio-Rad Laboratories, USA). The quantification was performed using ImageJ (National Institutes of Health, USA). β-actin was used as a control for equal loading, and the results are expressed as the optical density of the target protein/β-actin.

#### Peripheral blood phenotype

Whole blood was collected from piglets into BD Vacutainer^®^ EDTA tubes (Becton Dickinson, USA) and processed for flow cytometry. In brief, 25 µL of blood was mixed with 75 µL antibody mixture in fluorescence-activated cell sorting (FACS) buffer (consisting of 0.2% bovine serum albumin (BSA), 0.2% Sodium Azide, and 0.05% horse serum in PBS). The pre-titrated antibody panels used were as follows: Panel 1 (CD45-FITC, SWC3-PE, CD21-A647), Panel 2 (CD4-FITC, CD3-SPRD, CD8α-APC, SWC6-PE), detailed information regarding antibodies is shown in Table S3. Following a 20-min incubation in the dark at room temperature, erythrocytes were lysed by adding 900 µL of distilled water. After 5 s, 10 × Hanks balanced salt solution (HBSS) was added to bring the solution back to an isotonic state. Cells were fixed in paraformaldehyde with a final concentration of 1% in PBS (Ampliqon, Denmark) and stored in the dark at 4 °C overnight before flow cytometric acquisition. Flow cytometric analyses were conducted on a BD FACSCelesta™ flow cytometer (BD Biosciences, USA), and analyses of acquired samples were performed in the FACSDiva™ Software (BD Biosciences, USA). Single stained and fluorescence minus one (FMO) control were included for both panels. Absolute counting of cells was achieved by adding 25 µL of 123count eBeads™ Counting Beads (01-1234-42, Thermo Fisher Scientific, USA) diluted in 41 µL FACS buffer immediately before acquisition. The flow cytometry gating strategy is shown in Fig. S1 and Fig. S2.

#### Acute phase proteins

Plasma haptoglobin concentration was measured using a Phase Range Haptoglobin Assay Kit (TP-801, Tridelta Developments Ltd, Ireland). Plasma CRP and Pig-MAP concentrations were determined by particle enhanced immune turbidimetry, using Turbovet pig CRP and Turbovet Pig-MAP kits (Acuvet biotech, Zaragoza, Spain), respectively. The determinations were conducted using an ADVIA 1800® Chemistry System autoanalyzer (Siemens Medical Solutions, USA).

### Calculations and statistical analysis

The LOQs of the *faeG*, *eltB*, and *est-II* genes in the qPCR data were established by applying 5 gene copies per 2 μL DNA elution. Subsequently, the LOQ log copies/g sample was calculated based on the exact weight of the sample used for DNA extraction.

*GAPDH* and *ACTB* used in gene expression were confirmed to be suitable as reference genes based on GeNorm analysis of M and coefficient of variation values, demonstrating their stability as reference targets. Pre-processing, normalization using reference genes, and relative quantification of the gene expression data were performed using GenEx7 (MultiD, Göteborg, Sweden). The relative quantity of samples for each gene was calculated as relative to the lowest expression within the gene.

Statistical analyses were performed using R Studio (version 4.3.2) [21]. Model diagnostics were performed using the R packages DHARMa [22] and performance [23], except for the fecal score model, where the *appraise* function of the gratia package was used [24]. Principal component analysis (PCA) was performed using the *prcomp* function from the stats package on centered and scaled data, including systemic parameters monitored across all days, i.e., fecal DM and qPCR data, acute phase proteins and hematology data; and intestinal parameters on d 4, i.e., digesta DM and qPCR data, gene expression data, OCLN protein levels and morphology data. Prior to analysis, missing values were imputed as median treatment group levels on the day of sampling; either d 0 to 4 or post-sacrifice. Data visualization was performed using the ggplot2 package (v. 3.5.1).

For feces and blood samples collected at various time points, experimental group, day, and their interaction were included as fixed effects in all analyses. For samples from the digestive tract, experimental group, segment, and their interaction were included as fixed effects. For growth performance data [body weight, average daily gain (ADG), average daily feed intake (ADFI), and gain to feed ratio (G:F)], experimental group was the only fixed effect, with body weight at birth being a covariate in the analysis of ADG and ADFI. Repeated measurements were structured by including random effects in models, which accounted for samples collected on different days or from different gut segment samples from the same individual.

Linear mixed-effects models, implemented via the lme4 package [25], were used to analyze growth performance, DM content in fecal and digesta samples, cell count in blood, F4^+^ ETEC plate count, CRP and haptoglobin levels, gene expression data, OCLN protein levels, mucosa qPCR data, and rectal temperature. Generalized linear mixed models were used to analyze Pig-MAP level and qPCR data from feces and digesta, using the *glmer* function in the lme4 package. A hierarchical generalized additive model was used to analyze fecal score data using the mgcv package [26].

In Exp. A, data from d 0 post-weaning were excluded from the models analyzing F4^+^ ETEC levels in feces and fecal qPCR data. This was due to the initial baseline status of these data (d 0 was before the ETEC challenge so all the data were under the limit of detection), which resulted in minimal variance and subsequently caused issues with residuals in the model. In Exp. B, three pigs from the ETEC group were excluded from the dataset (one died on d 1, and the two others did not successfully receive the ETEC challenge). For the analysis of OCLN protein levels, one sample was identified as an outlier and consequently removed from the dataset.

Statistical comparisons between experimental groups were conducted using the emmeans package [27]. Significance levels were set at *P* < 0.05, with values 0.05 ≤ *P* < 0.10 indicating a tendency. To account for multiple comparisons, *P*-values from pairwise comparisons were adjusted using the Holm-Bonferroni adjustment method [28].

## Results

### BL1.2 and BL2.2 are compatible with feed processing and ad libitum in-feed provision

**Fig. 2:**
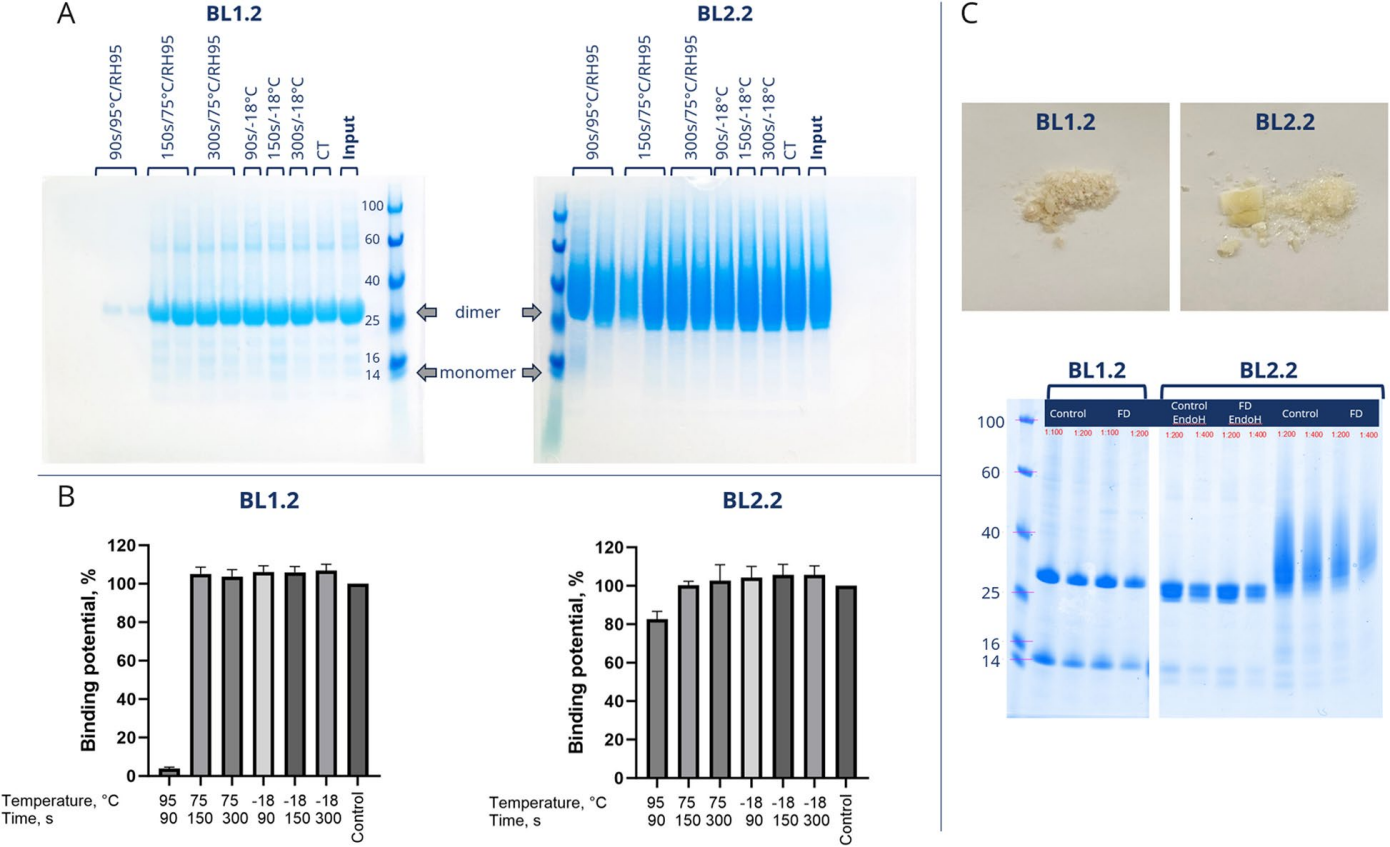
Characterization of V_H_H constructs and their stability upon freeze-drying and feed granulation.** A** SDS-PAGE showing V_H_H construct feed granulate stability after stability testing at various temperatures and time intervals, at 95% relative humidity for all samples above 0 °C, to emulate the storage and pelleting process. Arrows indicate expected migration of dimeric and monomeric V_H_H constructs. The analysis indicates that the dimeric V_H_H constructs possess high stability under all tested conditions, except for BL1.2 at 95 °C. **B** ELISA analysis of granulate samples, for quantitative relative stability of each sample measured as retained binding activity (compared to untreated granulate), the analysis confirms the SDS-PAGE. **C** Picture of final freeze-dried material for feed mixing (top). SDS-PAGE analysis showing the V_H_H construct integrity pre and post freeze-drying (FD), shown for BL1.2, BL2.2 after EndoH treatment for removal of N-glycosylation, and BL2.2 (bottom). The analysis indicates that the V_H_H constructs remain intact after freeze-drying, and that BL2.2 displays glycosylation

To evaluate the stability of the bivalent V_H_H constructs BL1.2 and BL2.2 under industrial feed processing conditions, V_H_H constructs were subjected to granulation and simulated feed pelleting conditions. The stability of V_H_H constructs was confirmed through analyses of protein integrity and binding activity (Fig. 2A and B), demonstrating high stability under exposure to differentiated temperature, relative humidity, and time. SDS-PAGE analysis showed that the freeze-dried V_H_H constructs to be used in the in vivo trial (Exp. A) retained good protein integrity (Fig. 2C).

In Exp. A, the freeze-dried V_H_H constructs were mixed into a mash feed and provided ad libitum to piglets at two different doses. During the trial, data were collected on body weight, feed intake, fecal samples, and blood samples. Daily intake of the V_H_H constructs increased from an average of 0.7 mg/V_H_H/pig the first two days to 40.2 mg/V_H_H/pig and 99.2 mg/V_H_H/pig by d 21 for the A-ETEC + BLlo and A-ETEC + BLhi groups, respectively (Table S4). The V_H_H constructs daily intake as part of the mash feed reached the intended levels by d 3 and 4 post-weaning: 5 mg/V_H_H/pig for A-ETEC + BLlo group and 15 mg/V_H_H/pig for A-ETEC + BLhi group.

**Fig. 3:**
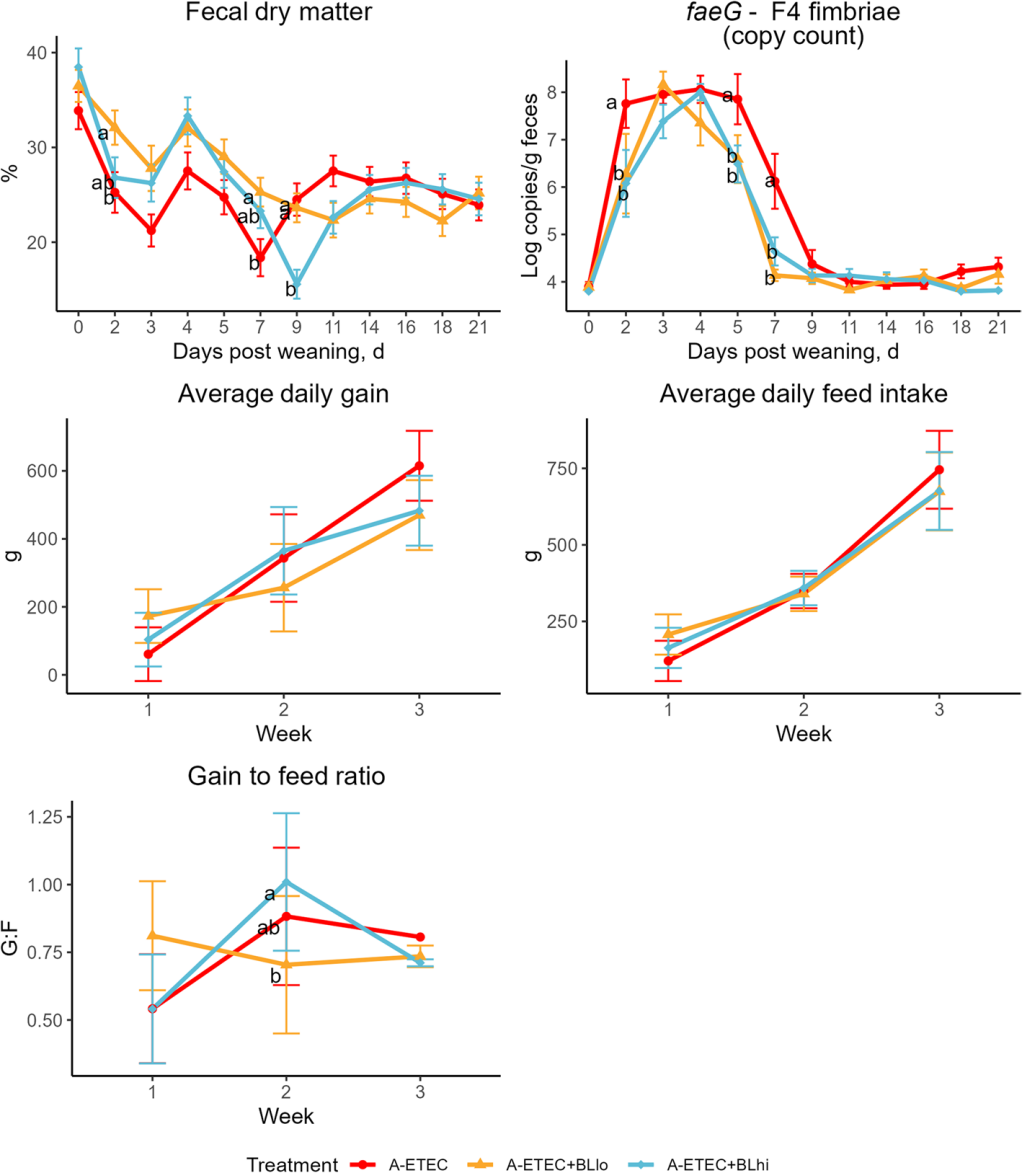
Effects of V_H_H constructs on fecal shedding, growth, and feed efficiency in Exp. A. Fecal levels of dry matter content, shedding of *faeG* (F4 fimbriae) in pigs, and the average daily gain, average daily feed intake and gain to feed ratio (G:F) across experimental groups. The limit of detection for fecal shedding of *faeG* gene level is 3.8 log copies/g sample. The F4^+^ ETEC was orally administered on d 1 and 2 post-weaning. Data are presented as estimated marginal means (emmeans) ± SE (except for F4^+^ ETEC and shedding of *faeG* gene data are presented as mean ± SE, with significance letter extracted from model). A-ETEC: challenged with ETEC, fed with non-binding V_H_H constructs (*n* = 10); A-ETEC + BLlo: challenged with ETEC, fed with 50 mg/kg feed V_H_H constructs (*n* = 10); A-ETEC + BLhi: challenged with ETEC, fed with 150 mg/kg feed V_H_H constructs (*n* = 10). ^a,b^Indicate statistical significance (*P* < 0.05) between experimental groups based on pairwise comparisons (Holm-Bonferroni adjustment). Groups sharing the same letter are not significantly different at the respective time point

Following the ETEC challenge on d 1 and 2, significant differences were observed in fecal consistency scores (*P* = 0.0005) and fecal dry matter contents (%) (*P* < 0.05) among the experimental groups, with variations across different days (Fig. S3, Fig. 3). A higher proportion of firm feces was observed in both groups A-ETEC + BLlo and A-ETEC + BLhi across week one, whereas a lower degree of firm feces was observed in week two post weaning, compared to the A-ETEC group. Most notably, F4^+^ ETEC shedding, F4 fimbriae gene *faeG*, LT toxin gene *eltB*, and STb toxin gene *est-II* were significantly lower in groups A-ETEC + BLlo and A-ETEC + BLhi on d 2, 5, and 7 post-weaning, compared to the A-ETEC group (*P* < 0.05) (Fig. 3, Fig. S4). In the A-ETEC group, *faeG* fecal shedding lasted 11 d, reaching its peak around d 4 post weaning, while groups A-ETEC + BLlo and A-ETEC + BLhi showed a shorter duration of *faeG* shedding, lasting 7 d. During the second week of the experiment, the A-ETEC + BLlo group showed lower G:F than the A-ETEC + BLhi group (*P* = 0.003). No other significant differences in growth performance parameters or acute phase proteins across groups were observed (Fig. 3, Table S5).

### In-feed provision of BL1.2 and BL2.2 sustains piglet gut health upon an ETEC challenge

Following the findings from Exp. A, where V_H_H constructs in mash feed reduced ETEC shedding, a second in vivo trial (Exp. B) was conducted to evaluate the impact of these V_H_H constructs when administered in pelleted feed. This experiment aimed to assess the effects of V_H_H constructs on ETEC colonization and the short-term local and systemic immune responses in piglets, including a comparison with a non-challenged group.

In Exp. B, the daily intake of each V_H_H construct as part of pelleted feed for the B-ETEC + BL group (intended level: 15 mg/pig/d) was 4.3 mg/pig/d before ETEC challenge, and 9.4 mg/pig/d during the four days following the challenge (Table S4).

The B-ETEC group generally showed impaired growth performance compared to the B-CTRL and B-ETEC + BL groups (Table S6). The B-ETEC group had significantly lower ADG than the B-CTRL group during the post-challenge period (*P* = 0.006). Additionally, the B-ETEC group had a significantly lower G:F than the B-CTRL group (*P* < 0.05), and tended to have a lower G:F compared to the B-ETEC + BL group (*P* < 0.10) over the study periods.

**Fig. 4:**
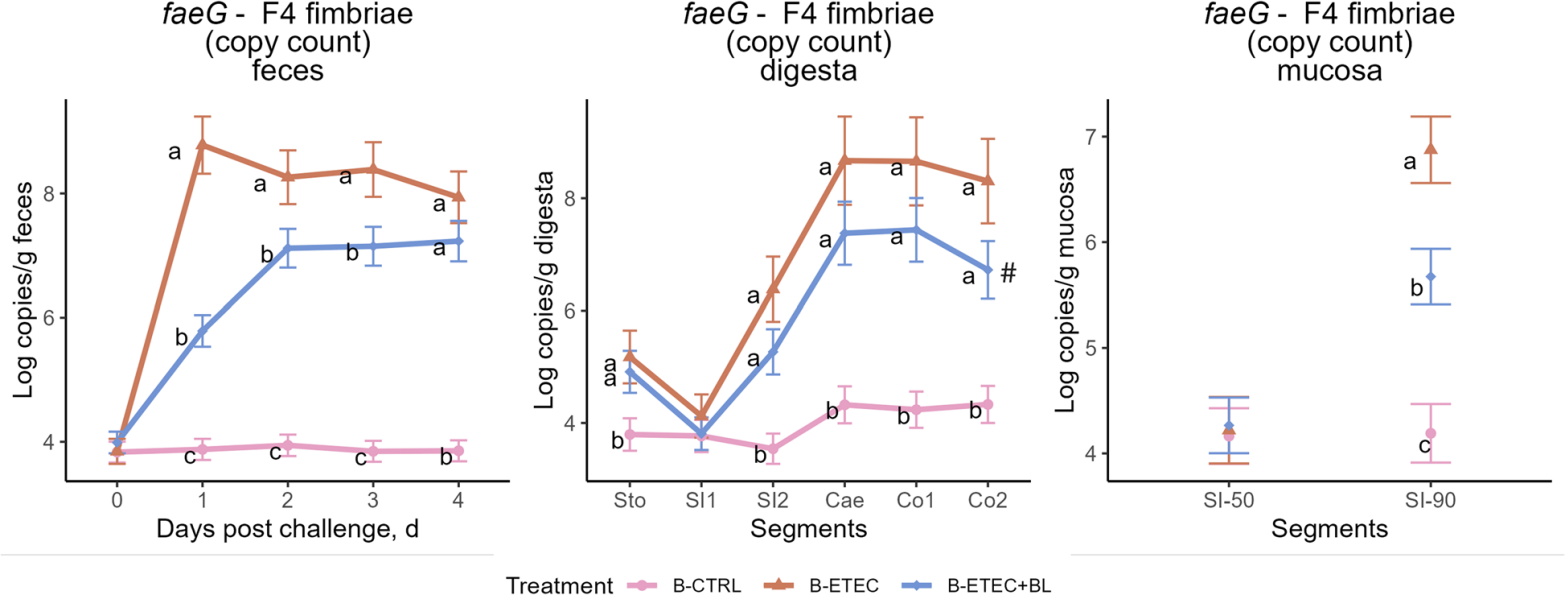
Levels of *faeG* (F4 fimbriae) gene (log copies/g) in pigs across experimental groups (Exp. B). The limit of detection for *faeG* gene level is 3.8 log copies/g sample. The F4^+^ ETEC was orally administered on d 0 and 1. Data are presented as estimated marginal means (emmeans) ± SE. B-CTRL: non-challenged, fed with control diet (*n* = 10); B-ETEC: challenged with ETEC, fed with control diet (*n* = 7); B-ETEC + BL: challenged with ETEC, fed with V_H_H constructs (*n* = 10). ^a–c^Indicate statistical significance (*P* < 0.05) between experimental groups based on pairwise comparisons (Holm-Bonferroni adjustment). Groups sharing the same letter are not significantly different at the respective time point or intestinal segment. ^#^The B-ETEC + BL group tended to show lower *faeG* level than the B-ETEC group in Co2 (*P* = 0.075)

**Fig. 5:**
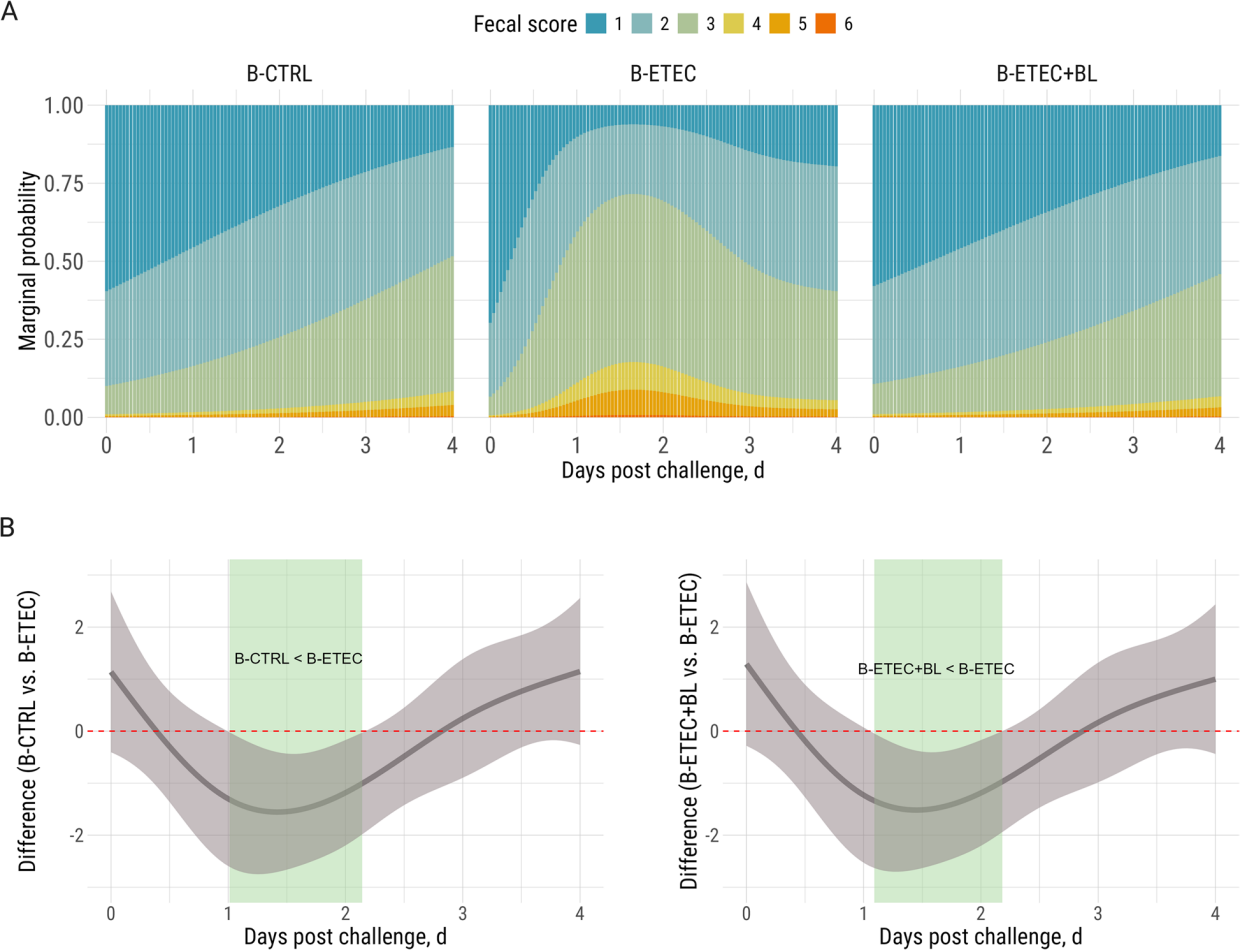
Fecal consistency scores in Exp. B: V_H_H-fed piglets display similar consistency to non-challenged piglets. The fecal consistency scores are based on a 7-point scale with scores 4–7 indicating diarrhea. Piglets were challenged with F4^+^ ETEC by oral administration on d 0 and 1. **A** Stacked bar plots for the marginal probability occurrence of each score category over time by experimental groups. **B** Differences between unchallenged piglets and ETEC-challenged piglets receiving a control diet (B-CTRL vs. B-ETEC, left) and differences between ETEC-challenged piglets receiving the bivalent V_H_H constructs in their feed and ETEC-challenged piglets receiving a control diet (B-ETEC + BL vs. B-ETEC, right). The graphs show that significant differences between the fecal consistency of these groups exist on d 1 and 2 post challenge, where the 95% empirical Bayesian simultaneous confidence interval does not cover 0 (green shade). B-CTRL: non-challenged, fed with control diet (*n* = 10); B-ETEC: challenged with ETEC, fed with control diet (*n* = 7); B-ETEC + BL: challenged with ETEC, fed with V_H_H constructs (*n* = 10)

The challenge was successful, as ETEC levels were detected in the feces of challenged piglets in the B-ETEC and B-ETEC + BL groups, but not in the B-CTRL group (Fig. 4). Daily supplementation of the bivalent V_H_H constructs significantly reduced ETEC levels both in feces and mucosa from distal small intestine (SI90), the latter seen as the B-ETEC + BL group had a lower ETEC fimbriae *faeG* level than the B-ETEC group (5.67 vs. 6.87 log copies/g, *P* = 0.005). The B-ETEC + BL group also tended to show a lower *faeG* level than the B-ETEC group in the distal colon (Co2) (*P* = 0.075). Our analysis further showed that piglets receiving the bivalent V_H_H constructs (B-ETEC + BL) had similar fecal DM content and fecal consistency scores to the non-challenged group (B-CTRL) (Fig. S5, Fig. 5). In contrast, the piglets that were challenged with ETEC but did not receive the bivalent V_H_H constructs (B-ETEC) had significantly lower fecal DM content on d 1 post-challenge. This was confirmed by the probability of diarrhea (scores 4 to 7) from d 1 to 2 being 13.6% in the B-ETEC group, 2.3% in the B-CTRL group, and 2.1% in the B-ETEC + BL group.

**Fig. 6:**
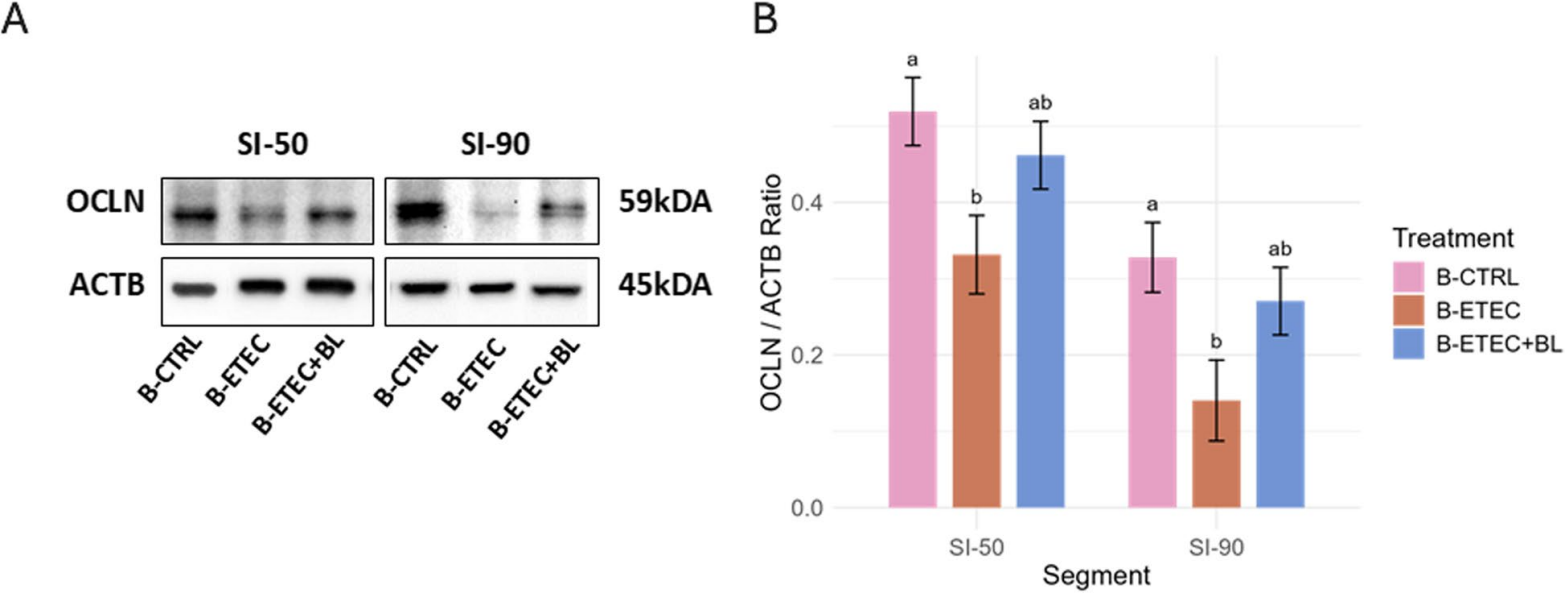
Western blot analysis of occludin (OCLN) levels in piglets across experimental groups in Exp. B. High OCLN levels are indicative of the gut epithelium being intact, whereas low OCLN levels can indicate that the piglets have a leaky gut. Data are normalized to the synthesis of the reference protein β-actin (ACTB) as relative intensity. Data are presented as **A** representative plot and **B** estimated marginal means (emmeans) ± SE plot. B-CTRL: non-challenged, fed with control diet (*n* = 10); B-ETEC: challenged with ETEC, fed with control diet (*n* = 7); B-ETEC + BL: challenged with ETEC, fed with V_H_H constructs (*n* = 10). ^a,b^Indicate statistical significance (*P* < 0.05) between experimental groups based on pairwise comparisons (Holm-Bonferroni adjustment). Groups sharing the same letter are not significantly different at the respective intestinal segment

Furthermore, to assess gut barrier function and health, OCLN levels were measured as a biomarker of gut epithelium permeability. The analysis revealed that B-ETEC + BL had OCLN levels intermediate between the B-ETEC and B-CTRL groups (Fig. 6). Morphological analysis of the small intestine showed lower (*P* < 0.01) villi/crypt (V/C) ratio in SI50, and tendency to lower (0.05 < *P* < 0.1) villi height in SI50 in B-ETEC and B-ETEC + BL groups compared to the B-CTRL group (Table S7), the representative images of H&E-stained sections are shown in Fig. S6.

### BL1.2 and BL2.2 modulate inflammatory responses in piglets upon an ETEC challenge

In Exp. B, blood and tissue samples were collected and analyzed to assess acute systemic and local immune responses to the ETEC challenge, and how these are impacted by supplementation with the bivalent V_H_H constructs. We quantified peripheral blood leukocytes and measured intestinal epithelial gene expression levels (Table 1, Fig. S7 and Fig. S8). Piglets in the B-ETEC group exhibited lower counts of blood granulocytes compared to the B-ETEC + BL group on d 1 to d 3 (*P* < 0.05) and to the B-CTRL group on d 3 to d 4 (*P* < 0.05) (Fig. S7). Granulocyte counts in the B-ETEC + BL were similar to those of the B-CTRL group at all sampling time points. Within the granulocyte population, neutrophil counts were significantly lower in the B-ETEC group compared to B-ETEC + BL on d 1 and d 2 (*P* < 0.05) (Table 1). In addition, the ETEC challenge decreased the eosinophil counts, while the provision of V_H_H constructs alleviated this decrease on d 2 and d 3.

**Table 1:** Complete blood cell counts of pigs in the experimental groups (Exp. B)^1^

**Item**	**Experimental group**^2^			**SEM**^3^	*	***P*-value**		
	**B-CTRL**	**B-ETEC**	**B-ETEC + BL**			**Group**	**Day**	**Group × Day**
Red blood cells, 10^12^ cells/L						0.03	< 0.001	< 0.001
d 0	6.72	6.78	6.83	0.13				
d 1	6.60	6.75	6.35	0.13				
d 2	6.39^b^	6.99^a^	6.12^b^	0.12				
d 3	6.18^b^	6.81^a^	6.24^b^	0.13				
d 4	6.33	6.56	6.20	0.15				
Hemoglobin, g/L						0.16	< 0.001	< 0.001
d 0	121	120.0	116	5				
d 1	118	120	107	5				
d 2	113^ab^	120^a^	104^b^	4				
d 3	108	117	104	4				
d 4	116	112	104	4				
Hematocrit, %						0.24	< 0.001	0.07
d 0	40.55	40.94	40.23	1.59				
d 1	39.28	39.39	36.44	1.56				
d 2	37.44	39.22	34.27	1.55				
d 3	36.14	39.29	35.26	1.57				
d 4	37.68	38.24	34.74	1.63				
Reticulocytes, 10^9^ cells/L						0.52	0.18	NS^4^
d 0	36.7	62.7	57.0	20.2				
d 1	43.8	74.8	68.1	24.2				
d 2	43.8	74.7	68.0	24.5				
d 3	32.0	54.6	49.7	18.3				
d 4	24.2	41.3	37.6	14.4				
Platelets, 10^9^ cells/L						0.94	0.02	NS
d 0	722	674	715	101	a			
d 1	695	649	688	97	ab			
d 2	661	618	655	92	ab			
d 3	636	594	629	88	ab			
d 4	641	599	635	89	b			
White blood cells, 10^9^ cells/L						0.15	0.42	0.01
d 0	11.6	15.5	13.4	1.2				
d 1	13.9	12.2	15.2	1.2				
d 2	13.8	10.4	14.2	1.2				
d 3	14.9	11.0	14.0	1.4				
d 4	15.5^a^	9.1^b^	13.0^ab^	1.5				
Neutrophils, 10^9^ cells/L						0.06	0.72	0.004
d 0	3.8	6.3	4.7	0.8				
d 1	4.5^ab^	3.4^b^	6.4^a^	0.8				
d 2	4.9^ab^	2.9^b^	6.7^a^	0.8				
d 3	6.2	3.7	6.0	1.0				
d 4	6.2	2.8	5.4	1.0				
Lymphocytes, 10^9^ cells/L						0.26	0.10	NS
d 0	7.3	8.0	7.8	0.6				
d 1	7.9	7.1	7.4	0.5				
d 2	7.6	6.7	6.1	0.5				
d 3	7.3	6.7	6.7	0.5				
d 4	7.8	5.9	6.3	0.6				
Monocytes, 10^9^ cells/L						0.15	< 0.001	< 0.001
d 0	0.10^b^	0.56^a^	0.76^a^	0.10				
d 1	0.75	0.70	0.73	0.14				
d 2	0.67	0.57	0.55	0.10				
d 3	0.67	0.55	0.75	0.11				
d 4	0.90^a^	0.42^b^	0.71^ab^	0.12				
Neutrophils/lymphocytes ratio						0.05	0.61	0.03
d 0	0.59	0.80	0.62	0.12				
d 1	0.57	0.48	0.88	0.11				
d 2	0.65^ab^	0.44^b^	1.11^a^	0.13				
d 3	0.86	0.57	0.91	0.15				
d 4	0.80	0.49	0.88	0.15				
Eosinophils, 10^9^ cells/L						< 0.01	0.02	0.07
d 0	0.26	0.19	0.18	0.05				
d 1	0.34^a^	0.13^b^	0.16^b^	0.05				
d 2	0.24^a^	0.07^b^	0.14^ab^	0.03				
d 3	0.27^a^	0.11^b^	0.20^ab^	0.04				
d 4	0.39^a^	0.11^b^	0.15^b^	0.05				
Basophils, 10^9^ cells/L						0.39	0.04	NS
d 0	0.003	0.003	0.005	0.002	b			
d 1	0.006	0.007	0.008	0.002	ab			
d 2	0.006	0.007	0.009	0.002	ab			
d 3	0.007	0.008	0.009	0.002	ab			
d 4	0.009	0.010	0.012	0.002	a			

^1^Values are presented as estimated marginal means (emmeans). d 0: the day of first ETEC challenge. Complete blood cell counts were measured using the IDEXX analyzer

^2^B-CTRL: non-challenged, fed with control diet (*n* = 10); B-ETEC: challenged with ETEC, fed with control diet (*n* = 7); B-ETEC + BL: challenged with ETEC, fed with V_H_H constructs (*n* = 10). The F4^+^ ETEC was orally administered on d 0 and 1

^3^Pooled standard error of least square means

^4^NS: not significant

^a,b^Values within a row without a common superscript indicate statistical significance (*P* < 0.05)

^*^For each item, values within a column without a common superscript differ between days (*P* < 0.05)

Gene expression analysis in the small intestinal epithelium revealed that the ETEC challenge tended to increase *TLR2* expression in both mid (SI50) and distal (SI90) small intestine compared to B-CTRL (*P* = 0.098), whereas the level in the B-ETEC + BL group was intermediate and did not differ significantly from either B-CTRL or B-ETEC groups (*P* > 0.1) (Fig. S8). Moreover, the ETEC challenge significantly increased the *IL10* expression compared to the B-CTRL group (*P* = 0.011), and this increase was not reduced by V_H_H constructs supplementation. The expression levels of *IL17A* and *IL23A* were very low across all samples (data not shown).

Further, we quantified levels of the acute phase proteins, CRP, haptoglobin, and Pig-MAP, in the experimental groups, which serve as indicators of inflammation. While the piglets in the B-ETEC group exhibited the highest levels of all three proteins (Table 2), the piglets fed with the bivalent V_H_H constructs (B-ETEC + BL) showed acute phase protein levels similar to the control piglets (B-CTRL), or intermediate levels between those of B-ETEC and B-CTRL.

**Table 2:** Plasma acute phase protein (CRP, Haptoglobin, Pig-MAP) concentrations in the experimental groups (Exp. B)^1^

**Item**	**Experimental group**^2^			**SEM**^3^	***P*-value**		
	**B-CTRL**	**B-ETEC**	**B-ETEC + BL**		**Group**	**Day**	**Group × Day**
C-reactive protein, μg/mL					0.05	< 0.001	< 0.001
d 1	0.02^b^	11.34^a^	5.11^a^	3.74			
d 2	2.41	2.39	1.5	1.41			
d 3	5.17	2.98	8.06	3.38			
d 4	9.43^A^	1.20^B^	7.67^B^	3.73			
Haptoglobin, mg/mL					0.02	< 0.001	< 0.001
d 0	0.7	0.9	0.7	0.2			
d 1	0.6^b^	2.5^a^	0.9^b^	0.3			
d 2	0.6^b^	2.2^a^	1.0^b^	0.3			
d 3	0.7^b^	1.7^a^	1.1^ab^	0.2			
d 4	1.1	1.1	1.3	0.3			
Pig-MAP, mg/mL					0.001	< 0.001	< 0.001
d 0	0.5	0.6	0.6	0.1			
d 1	0.5^c^	1.4^a^	0.8^b^	0.2			
d 2	0.5^b^	1.5^a^	0.9^a^	0.2			
d 3	0.7^b^	1.4^a^	1.1^ab^	0.2			
d 4	0.8	1.2	1.0	0.2			

^1^Values are presented as estimated marginal means (emmeans). d 0: the day of first ETEC challenge

^2^B-CTRL: non-challenged, fed with control diet (*n* = 10); B-ETEC: challenged with ETEC, fed with control diet (*n* = 7); B-ETEC + BL: challenged with ETEC, fed with V_H_H constructs (*n* = 10). The F4^+^ ETEC was orally administered on d 0 and 1

^3^Pooled standard error of least square means

^a–c^Values within a row without a common superscript indicate statistical significance (*P* < 0.05)

^A,B^Values within a row without a common superscript indicate a tendency to a difference (0.05 < *P* < 0.1)

### Multivariate analysis reveals an overall reduced systemic and intestinal response in ETEC-challenged piglets by in-feed BL1.2 and BL2.2

**Fig. 7:**
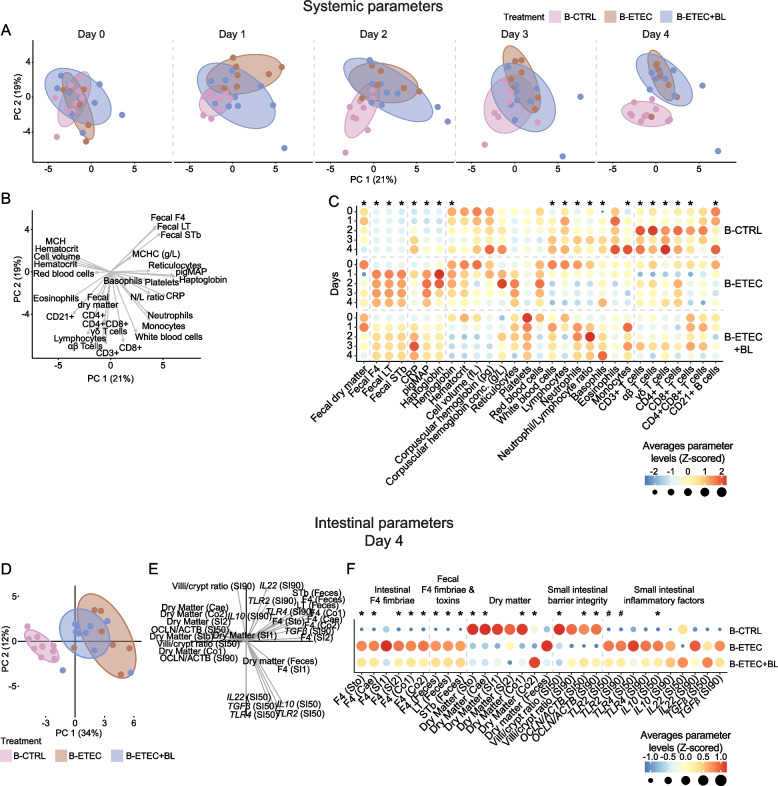
Multivariate analysis of systemic and intestinal parameters in Exp. B. Top: Systemic parameters. Fecal and systemic parameters from each treatment group (B-CTRL: *n* = 10, B-ETEC: *n* = 7, B-ETEC + BL: *n* = 10) collected at five timepoints: d 0 prior to initial treatment and four following days until pigs were sacrificed. **A** Score plots from a principal component analysis (PCA) performed on data from all timepoints. Data from each day were visualized individually to highlight temporal changes. Data were scaled and centered prior to analysis. Ellipses display the 80% confidence interval of each treatment group at each timepoint. **B** Loading plot. **C** Heatmap displaying average parameter levels for each treatment group at each timepoint. Average parameter levels were Z-scored across timepoints prior to visualization. ^*^Indicate statistical significance (*P* < 0.05) between experimental groups. Bottom: Intestinal parameters collected at day 4. **D** Score plot from PCA of intestinal parameters. Data were scaled and centered prior to analysis. Ellipses display the 80% confidence interval of each treatment group. **E** Loading plot. **F** Heatmap displaying average parameter levels for each treatment group on day 4. Average parameter levels were Z-scored prior to visualization. ^*^Indicate statistical significance (*P* < 0.05) between experimental groups. ^#^ Indicates a statistical tendency (0.05 ≤ *P* < 0.1) between experimental groups

A PCA was performed to visualize the temporal changes and clustering patterns of the experimental groups (B-CTRL, B-ETEC, and B-ETEC + BL) (Fig. 7A and B). On d 0, the three experimental groups clustered closely together, indicating the initial states were similar. By d 4, B-CTRL showed a clear separation from both B-ETEC and B-ETEC + BL along the PC2 (Fig. 7A). The loading plot (Fig. 7B) provided insight into the parameters contributing most to the observed variance between groups, made evidence that fecal F4 fimbriae and toxins were major contributors to the separation on the PC2 axis, with the lowest levels in the B-CTRL group (Fig. 7C). Similarly, immune cell populations, including CD3^+^, CD8^+^, and αβ T cells, were highest in B-CTRL. The inflammatory markers Pig-MAP and haptoglobin were strongly associated with PC1. Although these parameters contributed to a temporal drift of the B-ETEC and B-ETEC + BL towards the positive side of the PC1 axis, they did not contribute to the separation among experimental groups on d 4.

Focusing on the intestinal changes on d 4, the B-ETEC + BL group clustered between B-CTRL and B-ETEC along PC1 (Fig. 7D). The F4 fimbriae and toxins in both feces and digesta represented the major drivers for this change between the groups, with the highest levels in B-ETEC and the lowest in B-CTRL (Fig. 7E and F). On the contrary, the percentage of intestinal dry matter from various intestinal regions and the OCLN/ACTB ratio in the small intestine were highest in B-CTRL. This indicates lower fecal water content and stronger barrier integrity in the non-challenged B-CTRL piglets, which is intermediate in B-ETEC + BL, and poorest in B-ETEC (Fig. 7F). The intermediate position of B-ETEC + BL between the B-ETEC and B-CTRL groups suggests that the V_H_H constructs partially mitigated the effects of the F4^+^ ETEC challenge on intestinal parameters.

## Discussion

In this study, we evaluated the efficacy of bivalent V_H_H constructs (BL1.2 and BL2.2) targeting ETEC virulence factors as feed additives to manage ETEC-associated PWD in post-weaning piglets. Our results demonstrate that both mash and pelleted feed formulations containing these bivalent V_H_H constructs can reduce the incidence of diarrhea and fecal shedding of ETEC, even under varying intake levels. The use of BL1.2 and BL2.2 in mash feed, provided ad libitum, can reduce ETEC proliferation to a similar extent as previously shown using controlled oral gavage supplementation of these constructs [14, 15, 29]. This is notable, especially considering the inherently high variability in feed intake among newly weaned piglets, particularly during the first days after weaning. Feed inclusion levels as low as 50 mg per bivalent V_H_H construct per kg of mash feed seem sufficient to retain the desired effect. The V_H_H constructs also resulted in a faster clearance of fecal ETEC, seen as levels approaching the limit of detection in the A-ETEC + BLlo and A-ETEC + BLhi groups on d 7 instead of d 11 in the A-ETEC group. A shorter duration with high levels of pathogen shedding reduces the risk of transmission between animals, and thereby also the risk of PWD in the pen.

The higher proportion of firm feces (reduced occurrence of diarrhea) from d 1 to d 4 post challenge in both groups supplemented with V_H_H constructs compared to the A-ETEC group indicates that these V_H_H constructs could reduce the diarrhea symptoms in weaners during this period. Moreover, the higher occurrence of diarrhea in A-ETEC group from d 1 to d 4 post challenge aligned with elevated fecal shedding of F4^+^ ETEC, which is consistent with the results of Rhouma et al. [30], who reported that reduction of F4^+^ ETEC in fecal samples was associated with less diarrhea in ETEC-challenged piglets. Kim et al. [31] reviewed that diarrhea typically lasts from 1 to 5 days after ETEC infection of weaned piglets. However, we observed more soft feces in both groups with BL1.2 and BL2.2 supplementation around the second week post challenge, which is beyond the typical duration of experimentally ETEC-induced diarrhea, and which was not consistent with the gradual reduction of F4 fimbriae and enterotoxin levels. Our observations suggest the complexity of gut health, and further studies are needed to understand the factors influencing gut recovery after the clearance of the inoculated ETEC.

While a higher feed efficiency (G:F) was observed in the A-ETEC + BLhi group compared to the A-ETEC + BLlo, the two inclusion rates showed similar overall effects. Since the feed would be pelleted in Exp. B, we opted for the high inclusion rate (15 mg/V_H_H/pig/d) to ensure sufficient effect, accounting for possible damage to the V_H_H constructs during the pelleting process, which included heating up to 81 °C. Further, the peak of ETEC shedding occurred at around d 4 post challenge, which is in agreement with several ETEC challenge studies on piglets, reporting the peak of ETEC disease with the highest hemolytic coliform or F4^+^ gene levels in the feces to be on d 4–5 post challenge [30, 32, 33]. We therefore opted for d 4 to sacrifice and obtain samples from the gastrointestinal tract in Exp. B, hypothesizing the time point for the infection peak would be the best suited for evaluating differences in immune responses, particularly for inflammation levels and gut barrier function.

Furthermore, in Exp. B, we for the first time analyzed the F4 fimbriae *faeG* gene levels in both intestinal mucosa and digesta, whereas previous studies have focused on fecal samples. Our results demonstrated that the specific V_H_H constructs BL1.2 and BL2.2, when provided ad libitum in pelleted feed, significantly reduced F4 fimbriae gene levels in mucosa from the distal small intestine. This finding supports the hypothesized mode of action, whereby BL1.2 binds to ETEC F4 fimbriae, thus preventing adhesion to the intestinal mucosa.

The use of these V_H_H constructs in pelleted feed also showed a modulatory effect on both systemic and local immune responses caused by the ETEC challenge. Systemically, both the B-ETEC and B-ETEC + BL groups exhibited elevated CRP levels compared to the B-CTRL group on d 1 post challenge (Table 2). The levels were relatively low compared to the levels reported by Houdijk et al. [34] at three days after ETEC challenge (200 μg/mL in Houdijk study vs. < 20 μg/mL in the present study). This initial rise in CRP suggests an acute inflammatory response stimulated by the ETEC challenge. Furthermore, the B-ETEC group showed a marked increase in haptoglobin, reaching levels of 2.5 mg/mL, which is higher than the mean value of 0.58 mg/mL for 4-week-old piglets in commercial farms, as reported by Pineiro et al. [35]. This elevation is indicative of an infection response, aligning with the criteria reported by Yu et al. [36], who considered haptoglobin levels above 2 mg/mL as an indicator of infection in post-weaning piglets. Similarly, Pig-MAP levels in the B-ETEC group were elevated, being higher than the mean value of 0.92 mg/mL for this age group of piglets [35]. The addition of V_H_H constructs to the feed prevented an increase in haptoglobin and Pig-MAP levels after the F4^+^ ETEC challenge, suggesting that these V_H_H constructs, even when granulated and supplied as an additive in pelleted piglet feed, help maintain piglet gut health by ameliorating the inflammation caused by ETEC.

Regarding the systemic innate immune response to the F4^+^ ETEC challenge, flow cytometry analysis showed that both monocytes and granulocytes in blood decreased post challenge in the B-ETEC group, with granulocytes reacting faster than monocytes (Fig. S7). A decrease in these cell types in peripheral blood could indicate their migration to infection sites. This aligns with the innate immune system’s rapid response to infection [37]. The rise in acute phase proteins (CRP, haptoglobin, and Pig-MAP) as early as d 1 post challenge in our study further supports the activation of a rapid inflammatory response. In addition, we observed a decrease in circulatory T cells (CD3^+^) and B cells (CD21^+^) in the ETEC-challenged groups. This observation aligns with findings by Wöchtl et al. [38], who reported reduced leukocyte, T cell (CD3^+^), and B cell (CD79α^+^) numbers in the blood of newborn piglets 4–6 days following infection with Shiga toxin-producing *E. coli* O157:H7. In addition, in our study, the B-ETEC group showed a tendency for decreased CD4^+^CD8α^+^ counts compared to the B-CTRL group. These double-positive T cells in porcine blood are likely a heterogeneous subset, including effector memory T cells, as suggested by Okutani et al. [39]. It is worth noting that the addition of the V_H_H constructs seems to prevent the reduction in CD4^+^CD8 ^+^ counts, suggesting a protective effect of V_H_H constructs on the immature immune system of post-weaning piglets. A reduction of cytotoxic T cells (CD4-CD8α^+^) was observed in the B-ETEC group on d 1 post challenge, which aligns with Salmond et al. [40, 41], who demonstrated that LT toxin triggers peripheral apoptosis of CD8^+^ T cells in mice. This may partially explain why the piglets fed with V_H_H constructs did not experience a reduction in CD8^+^ T cells, indicating that the LT toxin was likely bound by the V_H_H constructs BL2.2.

Locally, in the gut, V_H_H constructs supplementation may contribute to the maintenance of gut barrier integrity by mitigating the damage caused by F4^+^ ETEC, as indicated by the preservation of the tight junction protein OCLN. In agreement with other in vivo studies [42–44], the current ETEC infection downregulated OCLN protein levels in the jejunal and ileal epithelium. F4^+^ ETEC triggers membrane damage in porcine intestinal epithelial cells by causing the delocalization of zonula occludens-1 (ZO-1), reducing OCLN amounts and dephosphorylating OCLN, thereby activating the paracellular secretion pathway, which is important in the development of diarrhea [45]. Although not statistically significant, V_H_H constructs supplementation numerically prevented a reduction of the OCLN to β-actin ratio caused by the challenge, resulting in an intermediate level between the B-CTRL and B-ETEC groups. Inhibition of the ability of F4^+^ ETEC to adhere to the epithelium and neutralization of the LT toxin by the supplementation of V_H_H constructs likely explains the observed beneficial impact in maintaining the integrity of tight junctions and epithelial barrier function. It should be noted that different methods have different sensitivities in assessing gut barrier integrity. While molecular-level changes in OCLN protein levels were observed, this effect was not reflected in morphological data (villi height and V/C ratio), which may suggest that such changes do not always translate into detectable structural differences.

For the local gene expression, we observed a tendency toward increased *TLR2* gene expression at both SI50 and SI90 locations in response to the ETEC challenge (Fig. S8), which suggests an activation of the immune response to F4^+^ ETEC. This finding is consistent with Luo et al. [46], who reported elevated *TLR2* gene expression levels in the jejunum after four hours of exposure to F4^+^ ETEC compared to a non-pathogenic *E. coli* strain. Previous studies have shown that the activation of *TLR2* could selectively rearrange *ZO-1* [47], upregulate *ZO-1* gene expression [48], upregulate the translation of ZO-1 and OCLN proteins [49], and improve intestinal epithelial barrier integrity. In addition, Kim et al. [48] reported that blocking of *TLR2* tended to decrease the *OCLN* mRNA levels. Therefore, the upregulation of *TLR2* in our study might indicate that the intestinal epithelial barrier integrity is being enhanced following damage caused by the ETEC infection. Further studies are needed to confirm the role of *TLR2* in the context of ETEC-induced gut damage. Piglets receiving the V_H_H constructs had an intermediate level of *TLR2* expression, indicating that the V_H_H constructs partially counteracted the ETEC challenge. In addition, some impact of the ETEC challenge on *IL10* expression was observed, to which the V_H_H constructs did not seem to have any influence. *IL10* is an anti-inflammatory cytokine and its signaling is associated with regulatory T cells (Tregs), playing a crucial role in modulating immune responses [50]. In the current study, the upregulation of *IL10* in the ETEC-challenged groups, which is consistent with other studies [51–53], suggests a regulatory environment, requiring Tregs to ameliorate inflammatory responses following the ETEC challenge [50]. However, it needs to be kept in mind that both *IL10* and *TLR2* changes were relatively small. Thus, it is difficult to conclude on its biological impact.

Although the current study was not designed to investigate the impact of the V_H_H constructs on growth performance as a main parameter, our results indicate that these constructs support piglet growth and nutrition uptake in the face of an ETEC challenge. Similar findings were reported in a study by Virdi et al. [54], where piglets fed a diet containing V_H_H-IgA against F4ac showed a higher weight gain than control piglets 5–11 d after F4^+^ ETEC challenge.

One major limitation of the current study is the controlled experimental setup, which does not fully replicate the complexities and variability of commercial farming environments. In Exp. A, piglets received a challenge dose of 5 mL of 10^9^ CFU ETEC/mL; this did not lead to notable diarrhea or clear infection signs, i.e., lack of difference in acute phase proteins. Therefore, we increased the challenge dose to 9 mL of 10^9^ CFU/mL on the first challenge day in Exp. B, aiming to elicit more pronounced infection signs and to better assess differences in inflammation. This higher infection load did allow us to observe more diarrhea, i.e., the B-ETEC group showed lower fecal dry matter than the A-ETEC group, and a more pronounced inflammatory response in Exp. B. However, it also resulted in the death of one piglet from the B-ETEC group, and other piglets displaying more severe symptoms than expected, and thus led us to reduce the dose on the second challenge day to 5.5 mL of 10^9^ CFU/mL. The balance between aiming for a realistic disease model that offers sufficient differentiating power for detecting differences in tested parameters and ensuring animal welfare can be hard to strike. Future studies should therefore aim to validate these findings in large-scale field trials to confirm their robustness and practicality. Additionally, although we have quantified tight junction protein levels, the absence of immunofluorescence or scanning electron microscopy data limits direct visualization of tight junction integrity. While this study focuses on the short-term effects of bivalent V_H_H constructs supplementation in piglet feed, its long-term impact on pig health and productivity remains to be explored.

## Conclusion

This study demonstrates that in-feed provision of the bivalent V_H_H constructs, BL1.2 and BL2.2, can mitigate some of the negative effects of ETEC infection in post-weaning piglets. Our findings indicate that feed admixed V_H_H constructs can reduce ETEC colonization in the small intestine and that these V_H_H constructs have a modulatory effect on the immune response caused by ETEC. In particular, our data show that the V_H_H constructs counteract the impact of the ETEC challenge on acute phase proteins, *TLR2* gene expression, tight junction integrity, and lymphocyte populations in blood. These results indicate that V_H_H constructs can be used to mitigate ETEC-mediated PWD and improve gut health in piglets, which, combined with their very high biophysical stability under industrially relevant feed manufacturing conditions, support their potential as feed additives that could find utility in modern pig production.

## Supplementary Information

Additional file 1. Supplementary material of In-feed provision of binding proteins sustains piglet gut health and mitigates ETEC-induced post-weaning diarrhea. Table S1. Starter diet feed composition (Exp. A). Table S2. Pellet feed composition (Exp. B). Table S3. Monoclonal antibodies used for immunolabelling in flow cytometry analysis. Table S4. The daily intake (mg) of each VHH constructs per pig in Exp. A and Exp. B. Table S5. Plasma acute phase proteins (CRP, Haptoglobin) concentration of pigs in the experimental groups (Exp. A). Table S6. Body weight, average daily gain, average daily feed intake, gain to feed ratio (G:F), and rectal temperature of pigs in the experimental groups (Exp. B). Table S7. Morphological parameters of the small intestine in pigs in the experimental groups on day 4 post-challenge (Exp. B). Fig. S1. Gating strategy for analysis of granulocytes, monocytes, and lymphocyte subpopulations (CD45^+^, CD21^+^) in blood samples. Fig. S2. Gating strategy for analysis of lymphocyte subpopulations (CD3^+^, αβ T cells, γδ T cells, CD4^+^, CD8^+^, CD4^+^CD8^+^ cells) in blood samples. Fig. S3. Fecal consistency scores in Exp. A (based on a 7-point scale with scores 47 indicating diarrhea). Fig. S4. Fecal levels of F4^+^ ETEC (log CFU/g), shedding of *est-II* (STb toxin), *eltB* (LT toxin) gene (log copies/g) in pigs and body weight across experimental groups (Exp. A). Fig. S5. Dry matter content in fecal and digesta samples, and levels of *eltB* (LT toxin), *est-II* (STb toxin) gene (log copies/g) in pigs across experimental groups (Exp. B). Fig. S6. Representative images of H&E-stained sections of the mid (SI50) and distal (SI90) small intestine in pigs across experimental groups (Exp. B). Fig. S7. Flow cytometry analysis of peripheral blood lymphocyte subsets in pigs across experimental groups (Exp. B). Fig. S8. Epithelium gene expression in piglets across experimental groups (Exp. B).

## Data Availability

The datasets used or analyzed during the current study are available from the corresponding author on reasonable request.

## Abbreviations

ACTB: Actin beta
ADFI: Average daily feed intake
ADG: Average daily gain
BSA: Bovine serum albumin
Cae: Cecum
Co1: Proximal half of the colon
Co2: Distal half of the colon
CRP: C-reactive protein
Ct: Cycle threshold
DM: Dry matter
emmeans: Estimated marginal means
ETEC: Enterotoxigenic* Escherichia coli*
FACS: Fluorescence-activated cell sorting
FMO: Fluorescence minus one
G:F: Gain to feed ratio
GAPDH: Glyceraldehyde-3-phosphate dehydrogenase
HBSS: Hanks balanced salt solution
H&E: Hematoxylin and eosin
HRP: Horseradish peroxidase
IL: Interleukin
KASP: Competitive allele specific PCR
LOQ: Limit of quantification
LT: Heat‐labile enterotoxin
OCLN: Occludin
PBS: Phosphate buffered saline
PBST: 1 × PBS with 0.05% Tween20
PCA: Principal component analysis
Pig-MAP: Pig major acute-phase protein
PWD: Post-weaning diarrhea
qPCR: Quantitative real-time polymerase chain reaction
SI1: Proximal half of the small intestine
SI2: Distal half of the small intestine
SI50: 50% Of the small intestine length
SI90: 90% Of the small intestine length
ST: Heat‐stable enterotoxin
Sto: Stomach
TGFb: Transforming growth factor beta
TLR: Toll-like receptor
Tregs: Regulatory T cells
V/C: Villi/crypt
V_H_H: Heavy chain variable domain
ZO-1: Zonula occludens-1
