# Supplementary material for: In-feed provision of binding proteins sustains piglet gut health and mitigates ETEC-induced post-weaning diarrhea
Source: J Anim Sci Biotechnol. 2025 Jun 2;16:78. doi: 10.1186/s40104-025-01209-6 (PMC12128348; doi:10.1186/s40104-025-01209-6)
Supplement: Supplementary file 1 — Additional file 1. Supplementary material of In-feed provision of binding proteins sustains piglet gut health and mitigates ETEC-induced post-weaning diarrhea. Table S1. Starter diet feed composition (Exp. A). Table S2. Pellet feed composition (Exp. B). Table S3. Monoclonal antibodies used for immunolabelling in flow cytometry analysis. Table S4. The daily intake (mg) of each VHH constructs per pig in Exp. A and Exp. B. Table S5. Plasma acute phase proteins (CRP, Haptoglobin) concentration of pigs in the experimental groups (Exp. A). Table S6. Body weight, average daily gain, average daily feed intake, gain to feed ratio (G:F), and rectal temperature of pigs in the experimental groups (Exp. B). Table S7. Morphological parameters of the small intestine in pigs in the experimental groups on day 4 post-challenge (Exp. B). Fig. S1. Gating strategy for analysis of granulocytes, monocytes, and lymphocyte subpopulations (CD45+, CD21+) in blood samples. Fig. S2. Gating strategy for analysis of lymphocyte subpopulations (CD3+, αβ T cells, γδ T cells, CD4+, CD8+, CD4+CD8+ cells) in blood samples. Fig. S3. Fecal consistency scores in Exp. A (based on a 7-point scale with scores 47 indicating diarrhea). Fig. S4. Fecal levels of F4+ ETEC (log CFU/g), shedding of est-II (STb toxin), eltB (LT toxin) gene (log copies/g) in pigs and body weight across experimental groups (Exp. A). Fig. S5. Dry matter content in fecal and digesta samples, and levels of eltB (LT toxin), est-II (STb toxin) gene (log copies/g) in pigs across experimental groups (Exp. B). Fig. S6. Representative images of H&E-stained sections of the mid (SI50) and distal (SI90) small intestine in pigs across experimental groups (Exp. B). Fig. S7. Flow cytometry analysis of peripheral blood lymphocyte subsets in pigs across experimental groups (Exp. B). Fig. S8. Epithelium gene expression in piglets across experimental groups (Exp. B). [file 40104_2025_1209_MOESM1_ESM.docx]

Supplementary Material

# Supplementary tables

**Table S1.** Starter diet feed composition (Exp. A).

| **Ingredients** | **%** |
| --- | --- |
| Wheat | 51.16 |
| Barley | 23.42 |
| ViloSoy, Soybean protein | 13.34 |
| Potato protein | 3.00 |
| Fish meal | 2.80 |
| Palm fatty acid distillate | 2.26 |
| Sugar beet molasses | 0.50 |
| Calcium carbonate | 0.76 |
| Monocalcium phosphate | 1.01 |
| Sodium chloride | 0.31 |
| Lysine sulphate 98 | 0.53 |
| Methionine DL98 | 0.12 |
| Threonine 98 | 0.15 |
| Tryptophan 99 | 0.05 |
| Valine L 96,5 | 0.06 |
| Vitamin premix | 0.40 |
| Ronozyme HiPhos | 0.03 |
| Luctarom Advance | 0.10 |

**Table S2**. Pellet feed composition (Exp. B).

| **Ingredient** | **%** |
| --- | --- |
| Wheat | 48.72 |
| Barley | 20.00 |
| Soybean meal, toasted | 6.50 |
| ViloSoy, Soybean protein | 7.44 |
| Potato protein | 4.00 |
| Whey powder Lactose 96 - 98% | 6.00 |
| Palm fatty acid distillate | 2.07 |
| Calcium carbonate | 1.24 |
| Monocalcium phosphate | 1.37 |
| Sodium chloride | 0.72 |
| Lysine sulphate 98 | 0.93 |
| Methionine DL98 | 0.19 |
| Threonine 98 | 0.21 |
| Tryptophan 99 | 0.07 |
| Valine L 96,5 | 0.08 |
| Vitamin premix | 0.40 |
| Ronozyme HiPhos | 0.02 |
| Seges Microgrits Black | 0.05 |

**Table S3.** Monoclonal antibodies used for immunolabelling in flow cytometry analysis.

| **Antibody Name** | **Clone** | **Specificity** | **Fluorochrome** | **Brand** | **Cat No.** |
| --- | --- | --- | --- | --- | --- |
| anti porcine CD45 FITC | K252.1E4 | All isoforms of CD45, leukocyte common antigen | FITC | BioRad | MCA1222F |
| anti porcine SWC3a/CD172a PE | 74-22-15 | Peripheral blood monocytes, granulocytes, dendritic cells | PE | Southern Biotech | 4525-09 |
| anti porcine CD21 A647 | BB6-11C9.6 | All mature circulating B lymphocytes | A647 | Southern Biotech | 4530-31 |
| anti porcine CD4α FITC | MIL17 | All Th lymphocytes in peripheral blood | FITC | BioRad | MCA1749F |
| anti porcine CD3e SPRD/PeCy5 | PPT3 | CD3 epsilon present on all T cells | SPRD/PeCy5 | Southern Biotech | 4510-13 |
| anti porcine CD8α APC | 76-2-11 | Subset of αβ TCR, γδ TCR, NK | APC | Southern Biotech | 4520-11 |
| anti porcine SWC6 PE | MAC320 | SWC6, expressed by most γδ TCR cells | PE | BD Biosciences | 561486 |

**Table S4.** The daily intake (mg) of each V_H_H constructs per pig in Exp. A and Exp. B.

| **Item** | **A-ETEC+BLlo** | **A-ETEC+BLhi** |
| --- | --- | --- |
| **Exp. A**^1^ | | |
| Concentration Days | 50 mg/kg | 150 mg/kg |
| d 1 | 0.05 | 0.00 |
| d 2 | 3.0 | 2.9 |
| d 3 | 5.2 | 8.1 |
| d 4 | 9.1 | 28.6 |
| d 5 | 12.5 | 33.1 |
| d 6 | 18.2 | 43.5 |
| d 7 | 24.4 | 55.4 |
| d 0 – d 7 | 10.4 | 24.5 |
| d 7 – d 14 | 17.0 | 53.8 |
| d 14 – d 21 | 33.7 | 101.4 |
| d 0 -d 21 | 20.4 | 59.9 |
| **Exp. B**^2^ | | |
|  | **B-ETEC+BL** | |
| Concentration Days | 52.5 mg/kg | |
| d –3 to d 0 | 4.3 | |
| d 0 to d 4 | 9.4 | |
| d –3 to d 4 | 7.3 | |

^1^In Exp. A, d 0: the day of weaning.

^2^In Exp. B, d –3: the day of weaning, d 0: the day of first ETEC challenge.

**Table S5.** Plasma acute phase proteins (CRP, Haptoglobin) concentration of pigs in the experimental groups (Exp. A)^1^.

| **Item** | **Experimental group**^2^ | | | **SEM**^3^ | ***** | ***P*-value** | | |
| --- | --- | --- | --- | --- | --- | --- | --- | --- |
|  | **A-ETEC** | **A-ETEC+BLlo** | **A-ETEC+BLhi** |  |  | **Group** | **Day** | **Group x Day** |
| **C-reactive protein, µg/mL** | | | | | | 0.14 | <0.001 | NS^4^ |
| d 0 | 8.7 | 15.8 | 9.6 | 4.8 | bc |  | | |
| d 3 | 4.3 | 7.9 | 4.8 | 2.3 | c |  |  |  |
| d 5 | 16.2 | 29.6 | 17.9 | 8.2 | b |  |  |  |
| d 7 | 94.4 | 172.2 | 104.2 | 46.8 | a |  |  |  |
| d 14 | 51.6 | 94.2 | 57.0 | 25.8 | a |  |  |  |
| **Haptoglobin, mg/mL** | | | | | | 0.48 | <0.001 | NS |
| d 0 | 0.5 | 0.3 | 0.2 | 0.2 | d |  | | |
| d 3 | 2.2 | 2.0 | 1.9 | 0.2 | a |  |  |  |
| d 5 | 1.6 | 1.4 | 1.3 | 0.2 | b |  |  |  |
| d 7 | 1.5 | 1.4 | 1.3 | 0.2 | b |  |  |  |
| d 14 | 1.2 | 1.0 | 0.9 | 0.2 | c |  |  |  |

^1^Values are presented as emmeans. d0: the day of weaning.

^2^A-ETEC: challenged with ETEC, fed with non-binding V_H_H constructs (*n*=10); A-ETEC+BLlo: challenged with ETEC, fed with 50 mg/kg feed V_H_H constructs (*n*=10); A-ETEC+BLhi: challenged with ETEC, fed with 150 mg/kg feed V_H_H constructs (*n*=10). The F4^+^ ETEC was orally administered on days 1 and 2 post-weaning.

^3^Pooled standard error of least square means.

^4^NS: not significant.

^*^For each acute phase protein, values within a column without a common superscript differ between days (*P* < 0.05).

**Table S6**. Body weight, average daily gain, average daily feed intake, gain to feed ratio (G:F), and rectal temperature of pigs in the experimental groups (Exp. B)^1^.

| **Item** | **Experimental group**^2^ | | | **SEM**^3^ | ***P*-value** | | |
| --- | --- | --- | --- | --- | --- | --- | --- |
|  | **B-CTRL** | **B-ETEC** | **B-ETEC+BL** |  | **Group** | **Day** | **Group x Day** |
| **Body weight, kg** | |  |  |  |  |  |  |
| d –3 | 7.2 | 7.3 | 7.2 | 0.1 | 0.68 | / | / |
| d 0 | 7.0 | 7.4 | 7.3 | 0.2 | 0.55 |  |  |
| d 4 | 8.0 | 7.4 | 7.7 | 0.3 | 0.32 |  |  |
| **Average daily gain, g** | |  |  |  |  |  |  |
| d –3 to d 0 | – 58 | 9 | 48 | 50 | 0.30 | / | / |
| d 0 to d 4 | 234^a^ | – 23^b^ | 115^†^ | 36 | 0.01 |  |  |
| d –3 to d 4 | 108^A^ | – 9^B^ | 86^A^ | 28 | 0.05 |  |  |
| **Average daily feed intake, g** | | |  |  |  |  |  |
| d –3 to d 0 | 45 | 70 | 84 | 19 | 0.25 | / | / |
| d 0 to d 4 | 197 | 109 | 183 | 29 | 0.14 |  |  |
| d –3 to d 4 | 133 | 90 | 141 | 24 | 0.37 |  |  |
| **Weight gain : feed intake** | |  |  |  |  |  |  |
| d –3 to d 0 | – 5.05 | 0.15 | 0.28 | 1.79 | 0.08 | / | / |
| d 0 to d 4 | 1.20^a^ | – 0.82^b^ | 0.59^‡^ | 0.39 | 0.02 |  |  |
| d –3 to d 4 | 0.81^a^ | – 0.47^b^ | 0.55^‡^ | 0.31 | 0.04 |  |  |
| **Rectal temperature, °C** | | | | | 0.07 | 0.28 | NS^4^ |
| d 0 | 38.7 | 38.7 | 39.1 | 0.1 |  |  |  |
| d 1 | 38.9 | 38.9 | 39.2 | 0.1 |  |  |  |
| d 2 | 38.8 | 38.8 | 39.2 | 0.1 |  |  |  |
| d 3 | 38.9 | 38.9 | 39.3 | 0.1 |  |  |  |
| d 4 | 38.9 | 38.9 | 39.2 | 0.1 |  |  |  |

^1^Values are presented as emmeans. d –3: the day of weaning, d 0: the day of first ETEC challenge.

^2^B-CTRL: non-challenged, fed with control diet (*n*=10); B-ETEC: challenged with ETEC, fed with control diet (*n*=7); B-ETEC+BL: challenged with ETEC, fed with V_H_H constructs (*n*=10). The F4^+^ ETEC was orally administered on days 0 and 1.

^3^Pooled standard error of least square means.

^4^NS: not significant.

^a,b^ Values within a row without a common superscript indicate statistical significance (*P*  <  0.05).

^A, B^ Values within a row without a common superscript indicate a tendency to a difference (0.05 < *P* < 0.1).

^†^ Indicates there is a statistical tendency in the parameter of the corresponding row when comparing B-ETEC+BL to B-CTRL, and B-ETEC+BL to B-ETEC.

^‡^ Indicates there is a statistical tendency in the parameter of the corresponding row when comparing B-ETEC+BL to B-ETEC, but there is no difference between B-ETEC+BL and B-CTRL.

**Table S7**. Morphological parameters of the small intestine in pigs in the experimental groups on day 4 post-challenge (Exp. B)^1^.

| **Item** | **Experimental group**^2^ | | | **SEM**^3^ | ***** | ***P*-value** | | |
| --- | --- | --- | --- | --- | --- | --- | --- | --- |
|  | **B-CTRL** | **B-ETEC** | **B-ETEC+BL** |  |  | **Group** | **Segment** | **Group x Seg** |
| **Villi height, µm** | | | | | | 0.20 | 0.65 | 0.08 |
| SI50 | 349^A^ | 263^B^ | 269^B^ | 24 |  |  |  |  |
| SI90 | 302 | 279 | 316 | 24 |  |  |  |  |
| **Crypt depth, µm** | | | | | | 0.14 | 0.04 | NS^4^ |
| SI50 | 255 | 273 | 286 | 12 | a |  |  |  |
| SI90 | 236 | 255 | 268 | 12 | b |  |  |  |
| **Villous height/crypt depth ratio** | | | | | | 0.003 | 0.33 | 0.049 |
| SI50 | 1.46^a^ | 0.99^b^ | 0.97^b^ | 0.10 |  |  |  |  |
| SI90 | 1.28 | 1.14 | 1.23 | 0.10 |  |  |  |  |
| **Villi density, No./mm** | | | | | | 0.36 | <0.001 | NS |
| SI50 | 4.5 | 4.7 | 4.5 | 0.2 | a |  |  |  |
| SI90 | 3.7 | 3.9 | 3.7 | 0.2 | b |  |  |  |
| **Crypt density, No./mm** | | | | | | 0.17 | 0.76 | NS |
| SI50 | 9.3 | 9.9 | 9.4 | 0.3 |  |  |  |  |
| SI90 | 9.2 | 9.9 | 9.3 | 0.3 |  |  |  |  |

^1^Values are presented as emmeans.

^2^B-CTRL: non-challenged, fed with control diet (*n*=10); B-ETEC: challenged with ETEC, fed with control diet (*n*=7); B-ETEC+BL: challenged with ETEC, fed with V_H_H constructs (*n*=10). The F4^+^ ETEC was orally administered on days 0 and 1.

^3^Pooled standard error of least square means.

^4^NS: not significant.

^a,b^ Values within a row without a common superscript indicate statistical significance (*P*  <  0.05).

^A,B^ Values within a row without a common superscript indicate a tendency to a difference (0.05 < *P* < 0.1).

^*^For each item, values within a column without a common superscript differ between SI50 and SI90. (*P* < 0.05).

# Supplementary figures

**
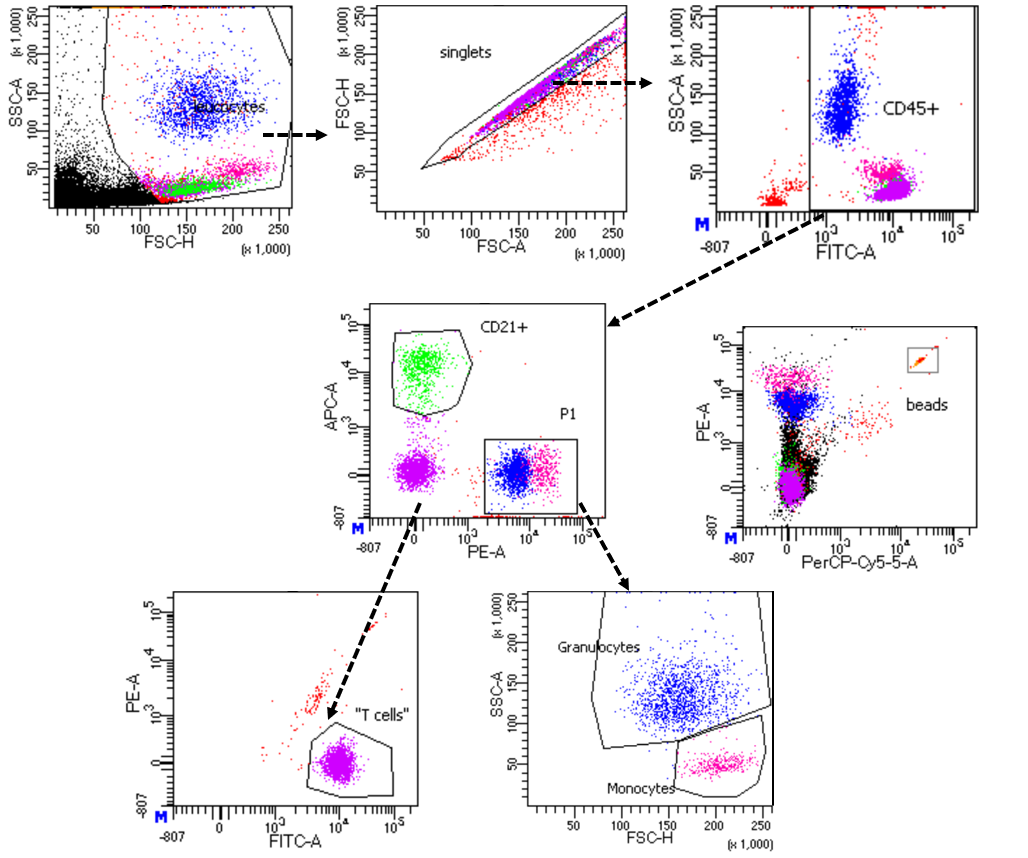
Fig. S1**. Gating strategy for analysis of granulocytes, monocytes, and lymphocyte subpopulations (CD45+, CD21+) in blood samples. Monocytes and granulocytes were determined through the exclusion of debris on forward scatter (FSC) and side scatter (SSC) dot plots, CD45+ gating using CD45-FITC-A and subsequently by FSC and SSC dot plots.


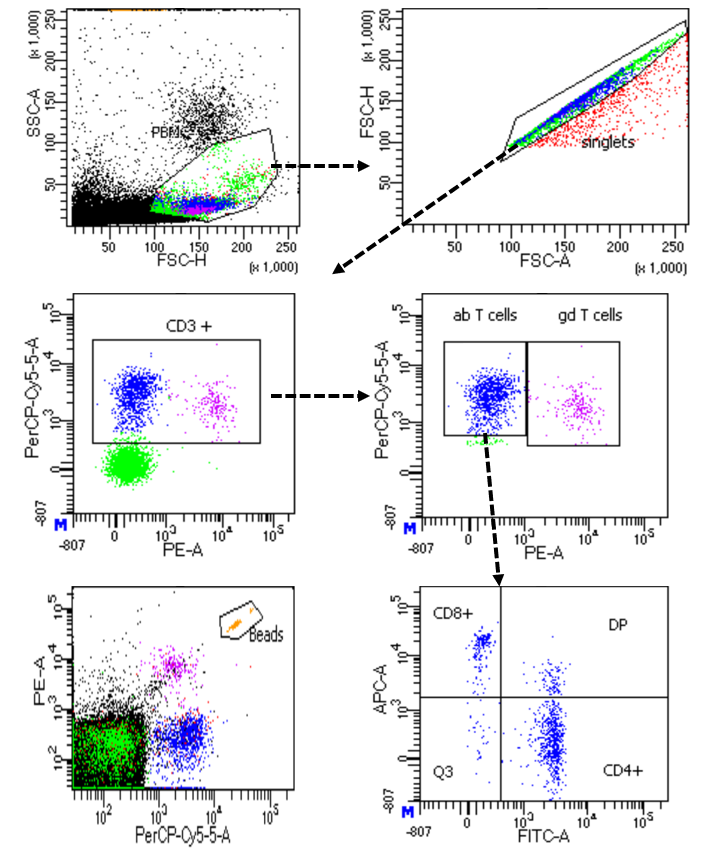
**Fig. S2**. Gating strategy for analysis of lymphocyte subpopulations (CD3+, αβ T cells, γδ T cells, CD4+, CD8+, CD4+CD8+ cells) in blood samples.


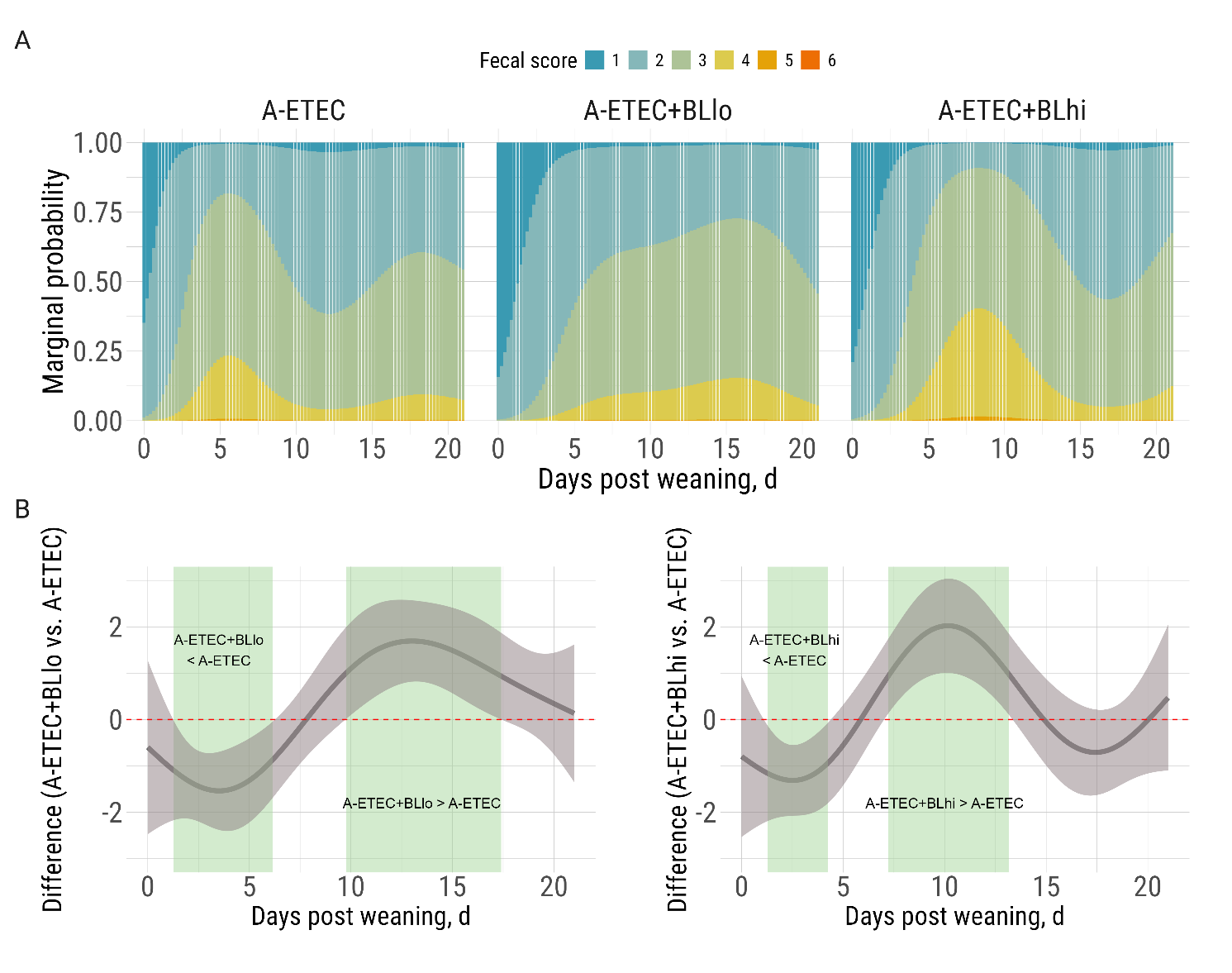
**Fig. S3**. Fecal consistency scores in Exp. A (based on a 7-point scale with scores 4-7 indicating diarrhea). Piglets were challenged with F4^+^ ETEC by oral administration on days 1 and 2 post-weaning. **A**) Stacked bar plots for the marginal probability occurrence of each score category over time by experimental groups. **B**) Differences between ETEC-challenged piglets receiving low level V_H_H constructs in their feed and ETEC-challenged piglets receiving a control diet (A-ETEC+BLlo vs. A-ETEC, left); and differences between ETEC-challenged piglets receiving high level V_H_H constructs in their feed and ETEC-challenged piglets receiving a control diet (A-ETEC+BLhi vs. A-ETEC, right). The graphs show that significant differences between the fecal consistency of these groups exist where the 95% empirical Bayesian simultaneous confidence interval does not cover 0 (green shade). A-ETEC: challenged with ETEC, fed with a control diet containing non-binding V_H_H constructs (*n*=10); A-ETEC+BLlo: challenged with ETEC, fed with 50 mg/kg feed V_H_H constructs (*n*=10); A-ETEC+BLhi: challenged with ETEC, fed with 150 mg/kg feed V_H_H constructs (*n*=10).

**
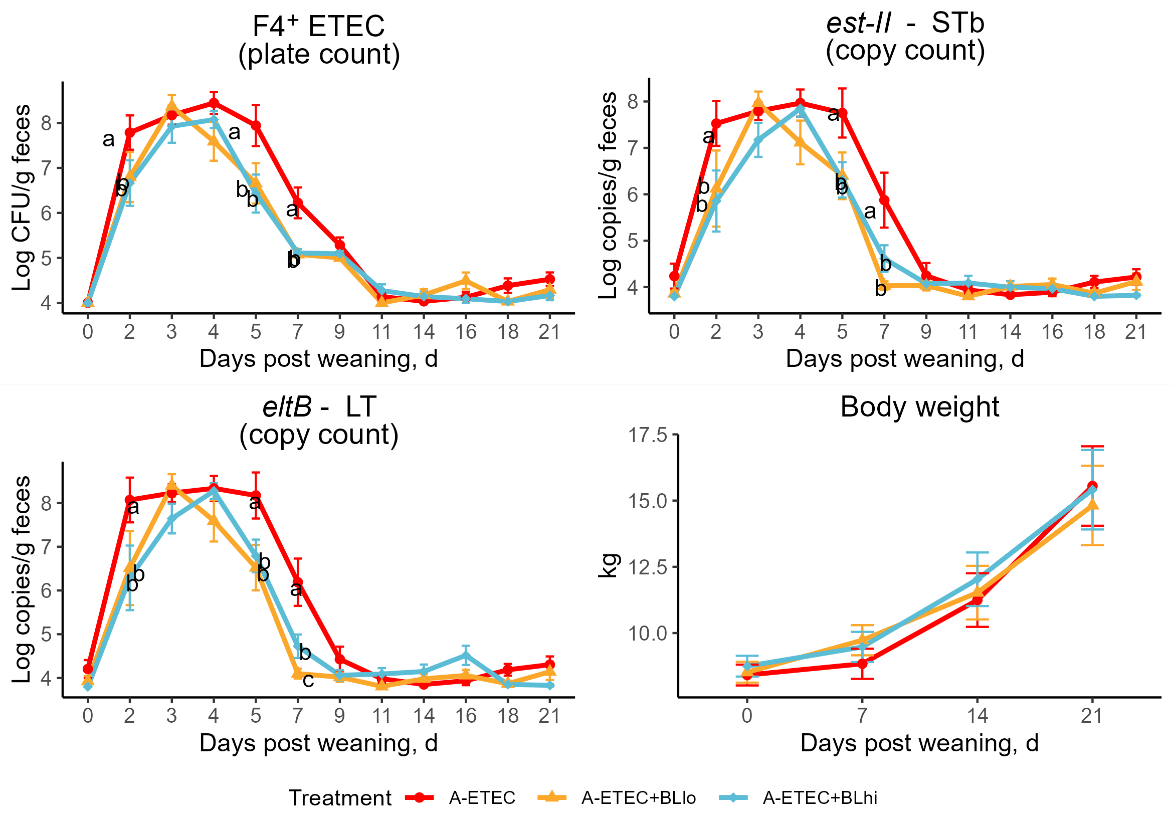
**

**Fig. S4**. Fecal levels of F4^+^ ETEC (log CFU/g), shedding of *est-II* (STb toxin), *eltB* (LT toxin) gene (log copies/g) in pigs and body weight across experimental groups (Exp. A). The limit of detection for F4^+^ ETEC level is 4 log CFU/g (except day 4 to 9 is 5 log CFU/g); the limit of detection for fecal shedding of *est-II*, *eltB* gene level is 3.8 log copies/g sample. The F4^+^ ETEC was orally administered on days 1 and 2 post-weaning. Data are presented as mean ± SE (except for body weight data are presented as emmean ± SE), with significance letter extracted from statistical model. A-ETEC: challenged with ETEC, fed with non-binding V_H_H constructs (*n*=10); A-ETEC+BLlo: challenged with ETEC, fed with 50 mg/kg feed V_H_H constructs (*n*=10); A-ETEC+BLhi: challenged with ETEC, fed with 150 mg/kg feed V_H_H constructs (*n*=10). ^a,b,c^ Indicate statistical significance (*P* < 0.05).


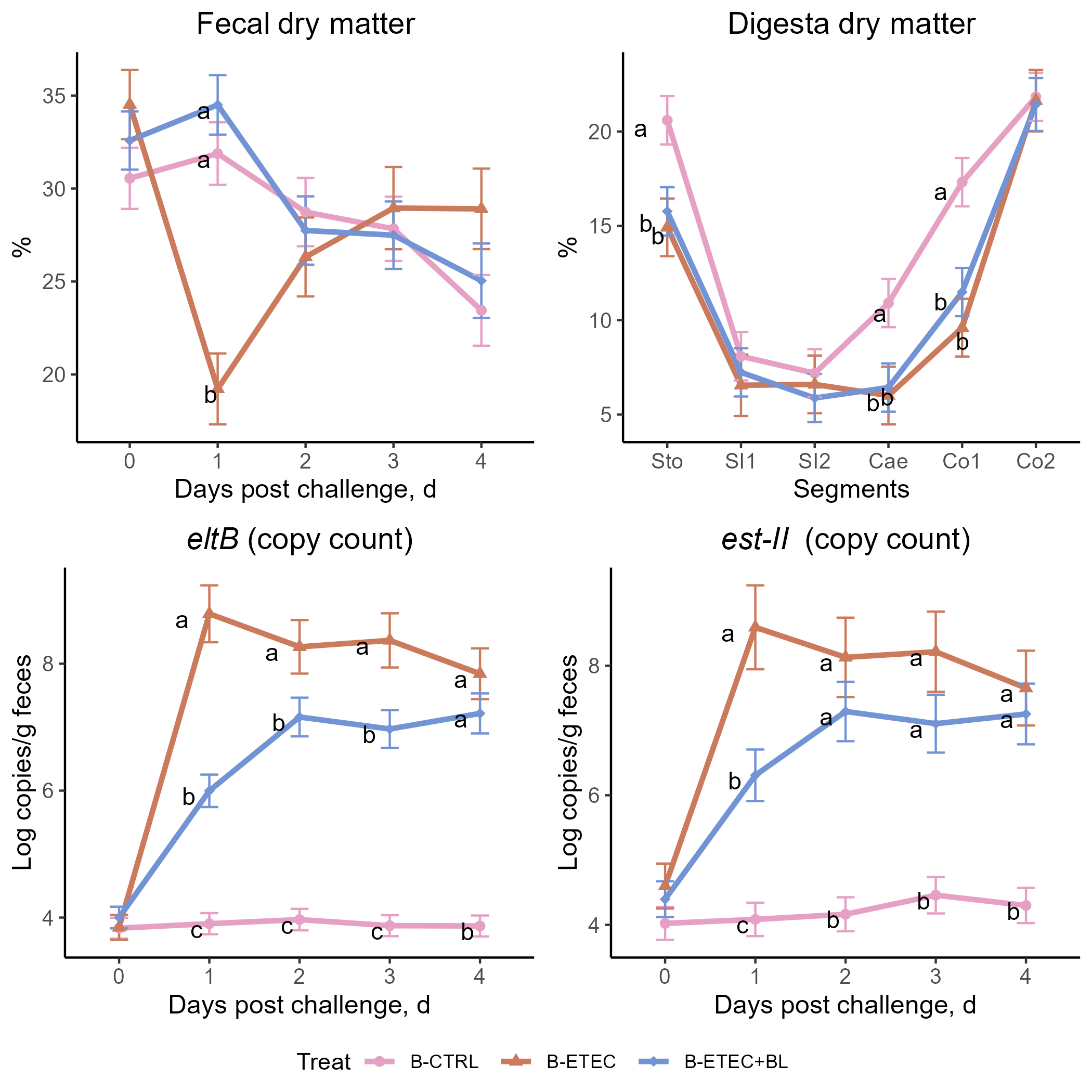


Fig. S5. Dry matter content in fecal and digesta samples, and levels of *eltB* (LT toxin), *est-II* (STb toxin) gene (log copies/g) in pigs across experimental groups (Exp. B).The limit of detection for *eltB*, *est-II* gene level is 3.8 log copies/g sample. The F4^+^ ETEC was orally administered on days 0 and 1. Data are presented as emmean ± SE. B-CTRL: non-challenged, fed with control diet (*n*=10); B-ETEC: challenged with ETEC, fed with control diet (*n*=7); B-ETEC+BL: challenged with ETEC, fed with V_H_H constructs (*n*=10). ^a,b,c^ Indicate statistical significance (*P* < 0.05).


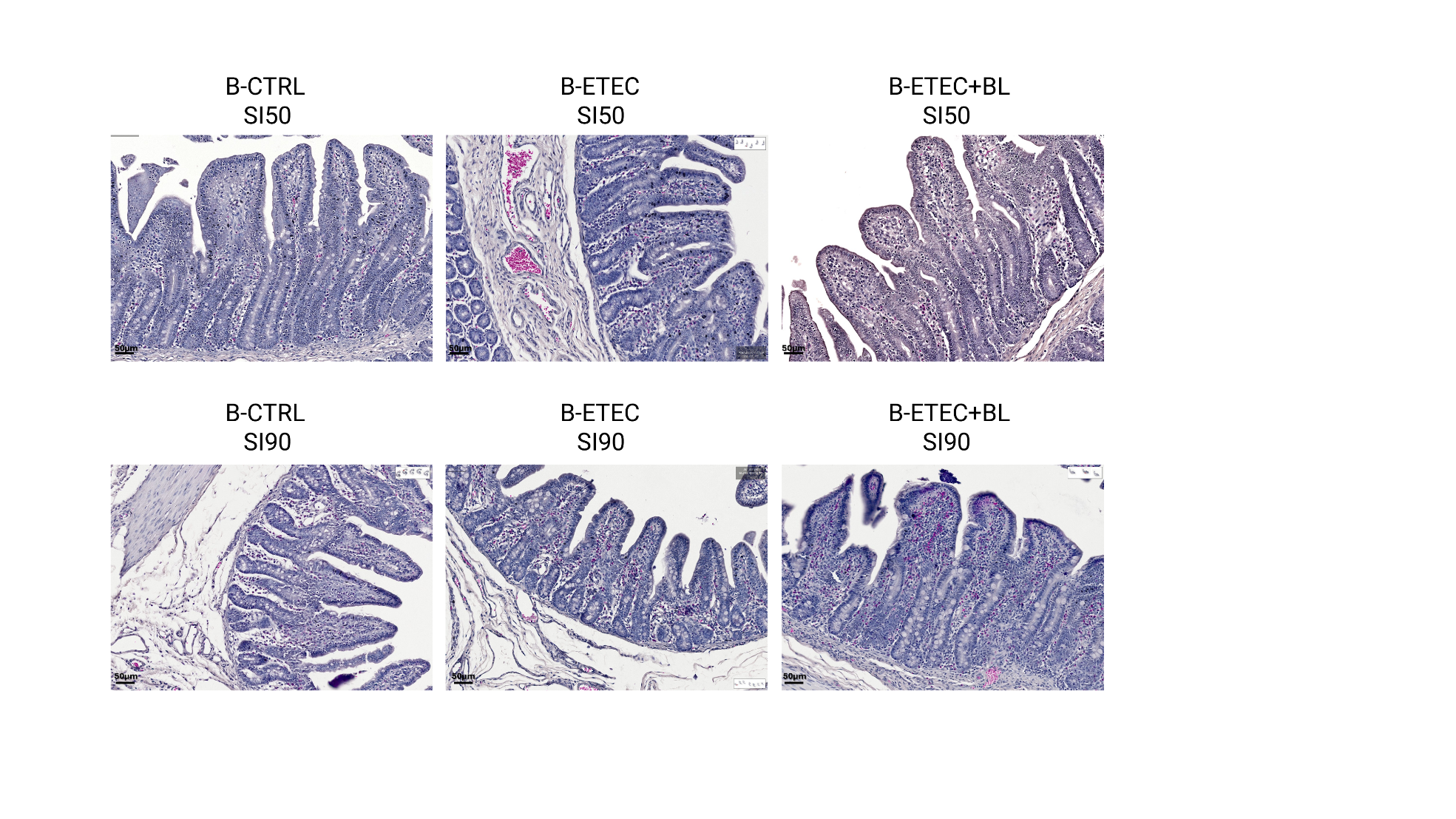
**Fig. S6**. Representative images of H&E-stained sections of the mid (SI50) and distal (SI90) small intestine in pigs across experimental groups **(**Exp. B**)**. Scale bar: 50 μm.

**
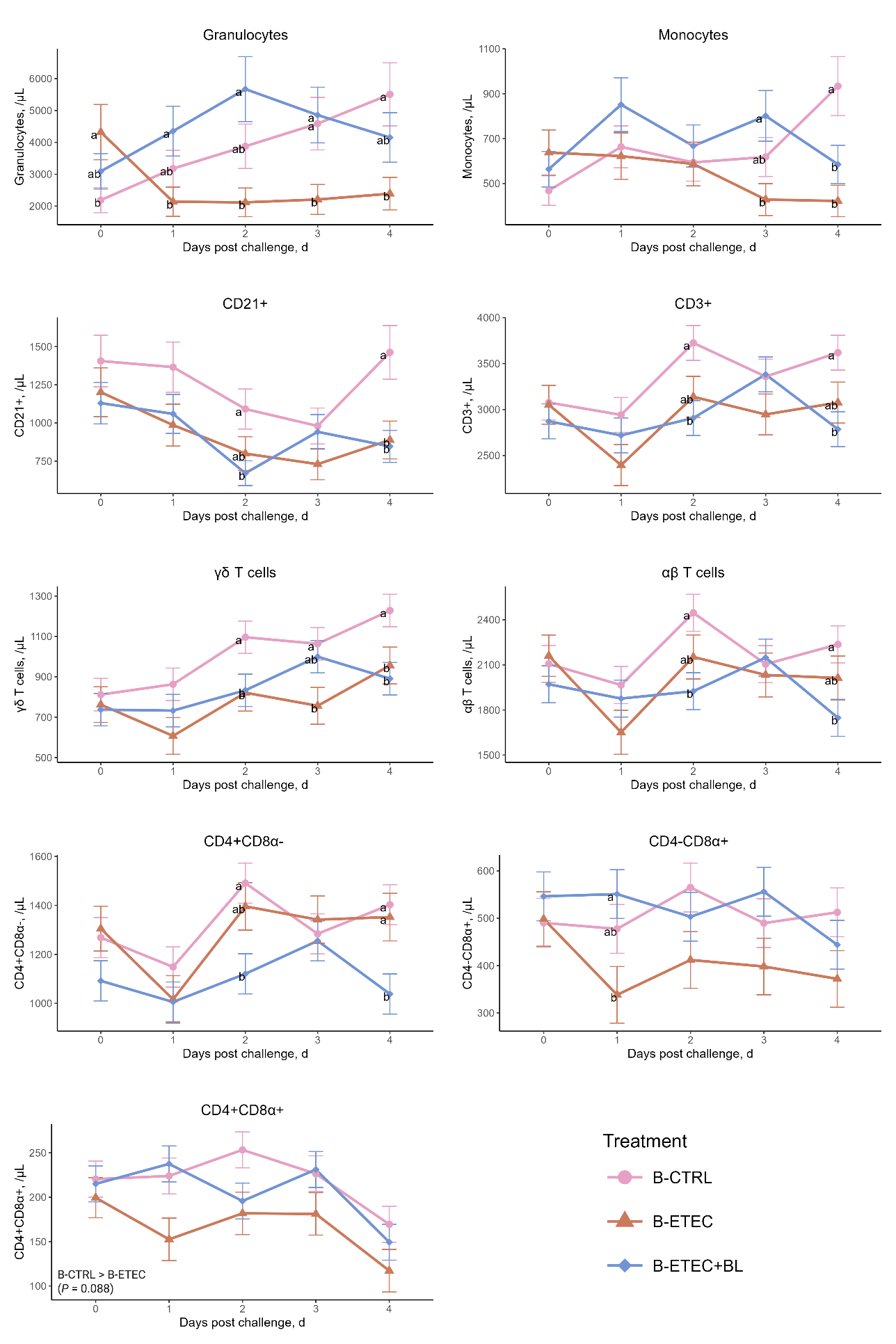
Fig. S7**. Flow cytometry analysis of peripheral blood lymphocyte subsets in pigs across experimental groups **(**Exp. B**)**. Each panel displays the quantification of granulocytes, monocytes, CD21+ B cells, CD3+ T cells, γδ T cells, αβ T cells, CD4+CD8α-, CD4-CD8α+, CD4+CD8α+ subsets. Data are presented as emmean ± SE. B-CTRL: non-challenged, fed with control diet (*n*=10); B-ETEC: challenged with ETEC, fed with control diet (*n*=7); B-ETEC+BL: challenged with ETEC, fed with V_H_H constructs (*n*=10). ^a,b^ Indicate statistical significance (*p* < 0.05).

**Fig. S8**. Epithelium gene expression in piglets across experimental groups (Exp. B). Fold-change relative to B-CTRL across groups and segments of *TLR2*, *TLR4*, *TGFb*, *IL22*, *IL10* are represented in the figures. Data are presented as emmean ± SE. B-CTRL: non-challenged, fed with control diet (*n*=10); B-ETEC: challenged with ETEC, fed with control diet (*n*=7); B-ETEC+BL: challenged with ETEC, fed with V_H_H constructs (*n*=10). ^a,b^ Indicate statistical significance (*p* < 0.05). ^A,B^ Indicate a statistical tendency between experimental groups (0.05 < *p* < 0.10).


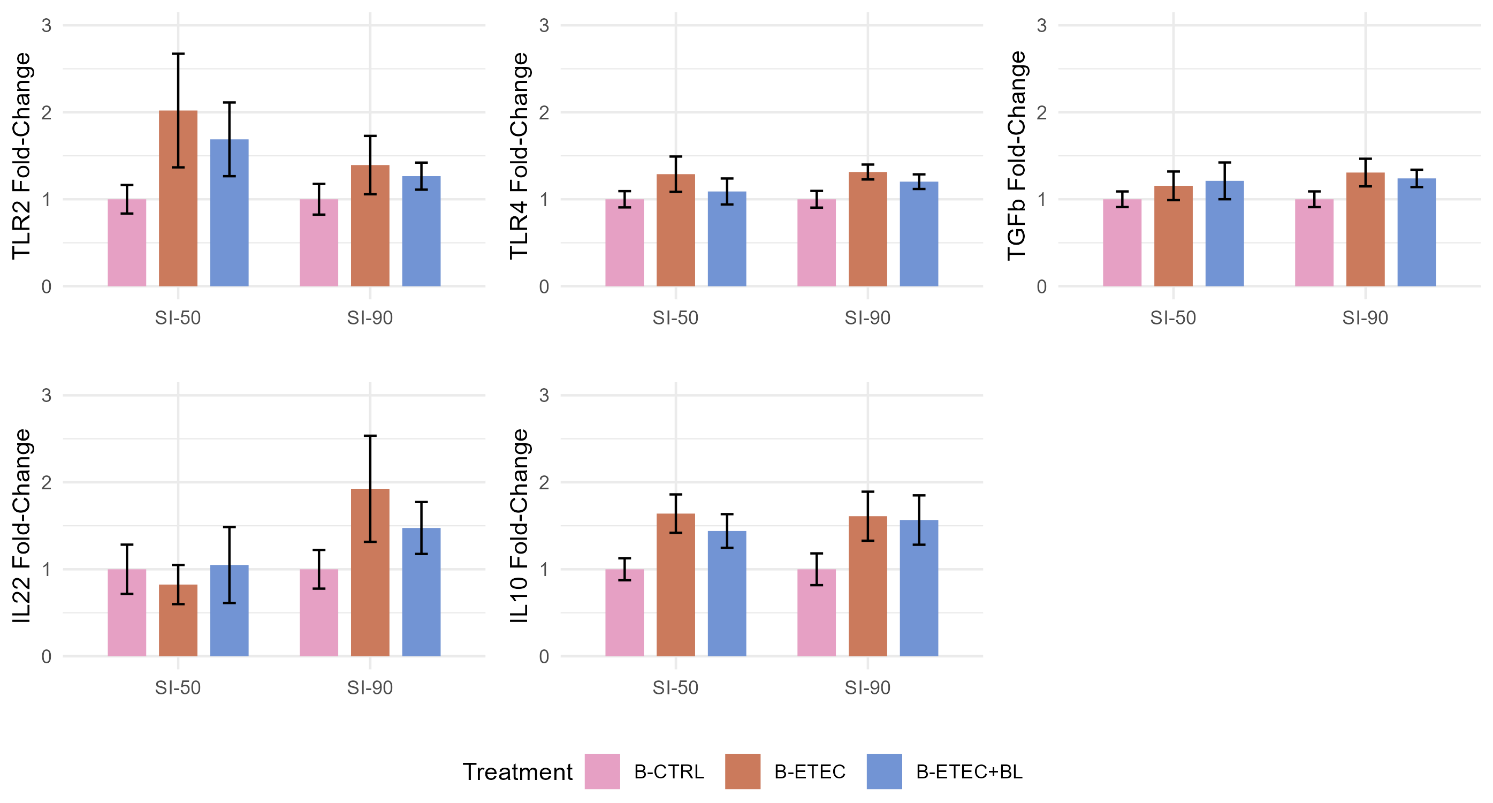


a

a

b

a a

b

A

AB

B

A

AB

B
